# Boosting hydrogen evolution on MoS_2_ via co-confining selenium in surface and cobalt in inner layer

**DOI:** 10.1038/s41467-020-17199-0

**Published:** 2020-07-03

**Authors:** Zhilong Zheng, Liang Yu, Meng Gao, Xiya Chen, Wu Zhou, Chao Ma, Lihui Wu, Junfa Zhu, Xiangyu Meng, Jingting Hu, Yunchuan Tu, Sisi Wu, Jun Mao, Zhongqun Tian, Dehui Deng

**Affiliations:** 1 State Key Laboratory of Physical Chemistry of Solid Surfaces, Collaborative Innovation Center of Chemistry for Energy Materials (iChEM), College of Chemistry and Chemical Engineering, Xiamen University, 361005 Xiamen, China; 2 State Key Laboratory of Catalysis, Collaborative Innovation Center of Chemistry for Energy Materials (iChEM), Dalian Institute of Chemical Physics, Chinese Academy of Science, 116023 Dalian, China; 3 School of Physical Sciences and CAS Key Laboratory of Vacuum Physics, University of Chinese Academy of Sciences, 100049 Beijing, China; 4 College of Materials Science and Engineering, Hunan University, 410082 Changsha, China; 5 National Synchrotron Radiation Laboratory, University of Science and Technology of China, 230029 Hefei, China

**Keywords:** Catalyst synthesis, Hydrogen energy, Electrocatalysis, Two-dimensional materials

## Abstract

The lack of highly efficient, inexpensive catalysts severely hinders large-scale application of electrochemical hydrogen evolution reaction (HER) for producing hydrogen. MoS_2_ as a low-cost candidate suffers from low catalytic performance. Herein, taking advantage of its tri-layer structure, we report a MoS_2_ nanofoam catalyst co-confining selenium in surface and cobalt in inner layer, exhibiting an ultra-high large-current-density HER activity surpassing all previously reported heteroatom-doped MoS_2_. At a large current density of 1000 mA cm^−2^, a much lower overpotential of 382 mV than that of 671 mV over commercial Pt/C catalyst is achieved and stably maintained for 360 hours without decay. First-principles calculations demonstrate that inner layer-confined cobalt atoms stimulate neighbouring sulfur atoms while surface-confined selenium atoms stabilize the structure, which cooperatively enable the massive generation of both in-plane and edge active sites with optimized hydrogen adsorption activity. This strategy provides a viable route for developing MoS_2_-based catalysts for industrial HER applications.

## Abstract (web summary)

The lack of efficient, inexpensive catalysts hinders large-scale application of hydrogen evolution reaction (HER). Here, the authors report a MoS_2_ nanofoam catalyst with co-confined Se in the surface and Co in the inner layer, exhibiting high large-current-density HER activity and durability.

## Introduction

The electrochemical splitting of water for producing hydrogen via renewable energy supply is a significant, carbon-neutral technology for clean energy generation^1–3^. However, the large-scale implementation of the reaction process is severely limited by scarcity of traditional platinum-based catalysts^4,5^. Two-dimensional MoS_2_, an earth-abundant material with unique structural and chemical properties, has shown attractive catalytic activity for the reaction and is considered as a potential alternative to the precious platinum-based catalysts in acidic medium^6–14^. However, only the edge S sites of pure MoS_2_ are catalytically active for the HER and the vast amount of S sites in the basal plane are quite inert and not sufficiently utilized^8,15–18^. Creating abundant edges is straight-forward but leads to high surface energy and low stability^15,19,20^. Doping metallic heteroatoms into the MoS_2_ lattice, on the one hand can effectively activate the basal plane S atoms and introduce in-plane active sites for the HER^10,21–25^, whereas on the other hand it may also unavoidably lead to over-activation of the edges, resulting in overly strong adsorption of hydrogen at the edge sites which is detrimental to the HER activity. Thus, selectively activating the inert basal plane combined with stabilizing the edges without quenching the activity can maximally increase active sites of MoS_2_ for the HER, but is highly challenging owing to the difficulty in balancing the activity and stability.

MoS_2_ possesses a tri-layer atomic structure, which allows multidimensional manipulation of atomic composition to modulate the catalytic activity. Herein, we report a strategy of co-confining Se in the surface and Co in the inner layer of the MoS_2_ lattice, which enables simultaneously activation of the basal plane and stabilization of the edges and optimization of the hydrogen adsorption activity. Combined with a morphology-controlling method of fabricating three-dimensionally nanofoam architecture to promote edge formation, the massive generation of both in-plane and edge active sites are achieved in the synthesized MoS_2_ nanofoam with co-confined Co and Se atoms (Co/Se-MoS_2_-NF). Such a catalyst exhibits a high HER activity at large current densities, which, to the best of our knowledge, surpasses those of all previously reported heteroatom-doped MoS_2_ catalysts and even the commercial precious Pt/C catalyst in acidic electrolyte. At a large current density of 1000 mA cm^−2^, the optimized Co/Se-MoS_2_-NF presents a much lower overpotential of 382 mV than that of 671 mV over the commercial Pt/C, and works stably for over 360 h without any activity decay. Density functional theory (DFT) studies demonstrate a synergy between the activating effect of confining Co in the inner Mo-layer and the stabilizing effect of confining Se in the surface S-layer, which promotes the formation of both in-plane and edge active sites and meanwhile optimizes the adsorption free energies of hydrogen. This strategy opens up a new prospect of tailoring the catalytic performance of MoS_2_ toward large-scale HER applications through confining multielements in different layers.

## Results

### Se-doping promotes edge formation

**Fig. 1:**
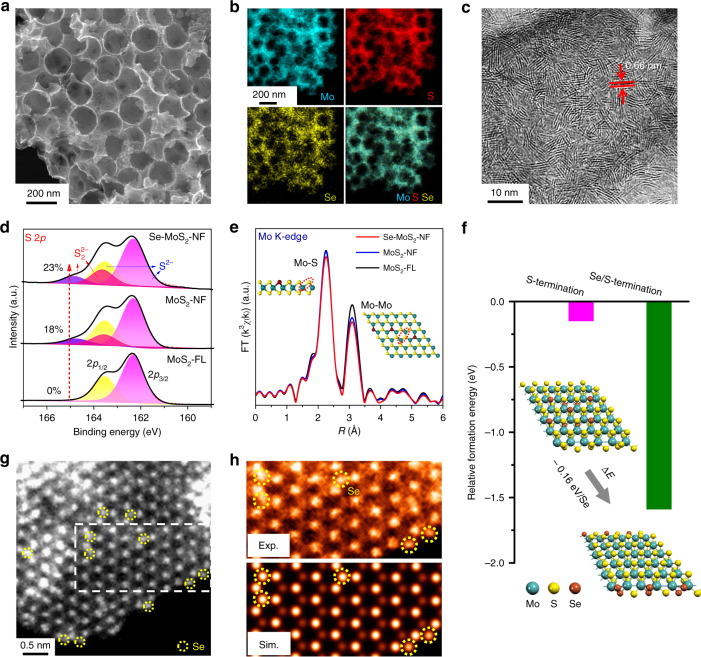
Structural and electronic properties of the Se-doped MoS_2_ nanofoam (Se-MoS_2_-NF). SEM image (**a**), EDX mappings (**b**), and HRTEM image (**c**) of the Se-MoS_2_-NF. Comparison in the decomposed XPS spectrum of the S 2*p* (**d**) and k^3^-weighted EXAFS spectra of Mo K-edge (**e**) between the Se-MoS_2_-NF, MoS_2_-NF and MoS_2_-FL catalysts. **f** Comparison in DFT calculated formation energies of the S-terminated and Se-terminated edges. **g** Low pass filtered large-scale atomic-resolution HAADF-STEM image of Se-MoS_2_-NF. The yellow doted circles denote the Se dopants. **h** Enlarged dotted region in **g** and the corresponding simulated image. The Se-MoS_2_-NF sample with 9.1% Se-doping content in atomic percentage was employed in these series of characterizations.

The terminating atoms of the MoS_2_ edges determine the stability and reactivity of the edges. Selenium being in the same group with S was introduced into MoS_2_ to modify the edge properties. A series of Se-doped MoS_2_ nanofoam (Se-MoS_2_-NF) with different Se content were synthesized via a one-pot solvothermal method by using (NH_4_)_6_Mo_7_O_24_·4H_2_O, CS_2_, and Se powder as precursors. Monodispersed SiO_2_ nanospheres were used as templates to fabricate a foam-like morphology. As shown in scanning electron microscopy (SEM) (Fig. 1a) and transmission electron microscopy (TEM) (Supplementary Fig. 1) images, the Se-MoS_2_-NF possesses a nanofoam morphology with abundant spherical cavities (average pore size ~100 nm), which will favor the mass transportation of reactants to access more active sites of the catalyst^15,19,21,26^.

Comprehensive spectroscopic characterizations of the Se-MoS_2_-NF samples were performed to investigate the geometric distribution and electronic states of the Se atoms. Energy-dispersive X-ray (EDX) analysis shows that the Mo, S, and Se elements are homogeneously distributed in the nanofoam framework (Fig. 1b). X-ray diffraction (XRD) analysis show no obvious difference in the crystallographic structure and lattice parameters of the Se-MoS_2_-NF compared with those of the pure MoS_2_-NF, which excludes the formation of new crystal phases after the Se doping (Supplementary Fig. 2 and Table 1). Raman spectroscopy of a Se-MoS_2_-NF sample displays a new satellite peak at the lower frequency side of the Mo-S vibrational peaks (Supplementary Fig. 3), which can be assigned to the A_1g_ mode of Mo-Se vibrations and thus indicates that Se atoms are embedded into the MoS_2_ lattice replacing the S atoms^27–30^. In the X-ray absorption near-edge structure (XANES) spectra of Se K-edge, the Se-MoS_2_-NF shows higher absorption edge than that in the MoSe_2_ (Supplementary Fig. 4), denoting a higher oxidation state of the Se atoms in the Se-MoS_2_-NF. It may be owing to the lower electronegativity of Se atom than that of S atom so that the Se atoms possess less charges in the Se-MoS_2_-NF^31–33^. These results demonstrate that the Se atoms are covalently doped into the MoS_2_ lattice rather than forming a new phase of MoSe_2_ or being adsorbed on the surface.

Se-doping combined with fabrication of nanofoam morphology leads to massive generation of edges. High resolution TEM (HRTEM) images show that the Se-MoS_2_-NF possesses abundant edges compared with the MoS_2_ nanofoam (MoS_2_-NF) and few-layer MoS_2_ (MoS_2_-FL) (Fig. 1c and Supplementary Fig. 5). The clear crystal lattice fringes with an interplanar spacing of 0.66 nm denotes the (002) planes of the 2H phase of MoS_2_. X-ray photoelectron spectroscopy (XPS, Fig. 1d) characterizations show that a new doublet peak arises at around 164.9 and 163.7 eV for the Se-MoS_2_-NF and MoS_2_-NF samples, which can be assigned to the bridging disulfide (S_2_^2−^) ligands at the edges^34–37^. This confirms that establishing a nanofoam morphology is favorable for the formation of more edges. In addition, the S_2_^2−^ content of 23% in the Se-MoS_2_-NF sample obtained from the peak area is higher than that of 18% in the MoS_2_-NF, thus implying that Se-doping further promotes the edge formation. This is also reflected by the extended X-ray absorption fine structure (EXAFS) spectroscopy of the Mo K-edge of these samples, which exhibits reduced Mo–Mo coordination of the Se-MoS_2_-NF compared with those of the MoS_2_-NF and MoS_2_-FL (Fig. 1e), thus indicating a decreased lateral sizes and increased exposure of edges in the Se-MoS_2_-NF^21,26,38^. DFT calculations also show that Se-saturated edge is more stable than S-saturated edge by 0.16 eV per Se replacing a S atom (Fig. 1f). These results demonstrate that Se-doping can promote the formation of abundant edges via stabilizing the edges. The Se-dopants were directly observed in the atomic-resolution high-angle annular dark field scanning transmission electron microscopy (HAADF-STEM) images of the Se-MoS_2_-NF, showing the homogeneous dispersion of the confined Se atoms in the basal plane and at the edge of the Se-MoS_2_-NF (Fig. 1g). The magnified image in Fig. 1h clearly illustrates that the S atoms are substituted by the Se atoms in accordance with the simulated image of the atomic model.

### HER performance of the Se-MoS_2_-NF

**Fig. 2:**
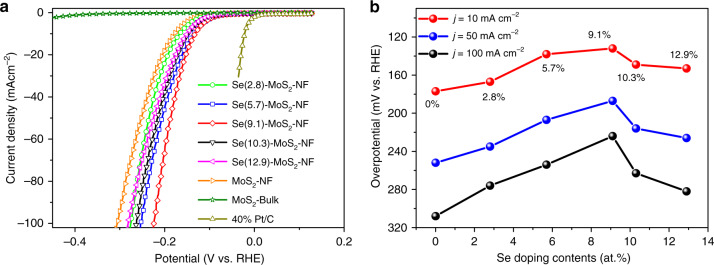
Electrocatalytic HER performances of the Se-MoS_2_-NF samples. **a** HER polarization curves for the Se-MoS_2_-NF with different Se doping contents in comparison with those of bulk MoS_2_, MoS_2_-NF, and 40% Pt/C. **b** Variation of the overpotentials depending on the Se doping content at current densities of 10, 50, and 100 mA cm^−2^, respectively. All catalysts are drop-casted on glassy carbon electrode for HER measurements, which were conducted in a 0.5 M H_2_SO_4_ electrolyte at 25 °C.

The Se-MoS_2_-NF samples present obviously enhanced HER activity compared with those of bulk MoS_2_, MoS_2_-FL, and MoS_2_-NF (Fig. 2a). The sample with Se doping content of 9.1% (Se(9.1)-MoS_2_-NF) exhibits the highest HER activity with overpotentials of 132, 187, and 224 mV at current densities of 10, 50, and 100 mA cm^−2^, respectively (Fig. 2b). The decrease in activity as the Se doping content exceeds 9.1% may be resulted from the significantly weakened adsorption of H* at the over-doped Mo-edge Se sites with much positive adsorption free energies (Supplementary Fig. 6). The slopes of linear part of the Tafel plots were used to investigate the HER mechanism over these catalysts. The introduction of Se atoms leads to slight decrease in the Tafel slope of MoS_2_ ranging from 75 to 80 mV dec^−1^, which indicates a Volmer–Heyrovsky mechanism (Supplementary Fig. 7). From the electrochemical impedance spectroscopy (EIS) of the electrode kinetics, the Nyquist plots (Supplementary Fig. 8 and Table 2) reveal a remarkable decrease in the charge-transfer resistance (*R*_ct_) from 44.1 Ω of the MoS_2_-NF to 11.6 Ω of the Se(9.1)-MoS_2_-NF, demonstrating the prominently improved interfacial electron-transfer kinetics over the Se(9.1)-MoS_2_-NF. Durability tests show that the HER performance of the Se(9.1)-MoS_2_-NF is well maintained for over 30 h at a current density of 10 mA cm^−2^, whereas those of the MoS_2_-NF and Pt/C (40 wt.%) drop rapidly (Supplementary Fig. 9). These results demonstrate the positive effect of Se doping in enhancing both the HER activity and stability of the MoS_2_.

### Properties of Co/Se-codoped MoS_2_

Substituting the Mo with Co atoms can effectively activate the adjacent S atoms and improve markedly the HER performance over the MoS_2_ basal plane^10,21,24^. To combine the activating effect of Co-doping with the effect of Se-doping in enriching the edges and boosting the HER activity, we designed a series of Co/Se-codoped MoS_2_ nanofoam (Co/Se-MoS_2_-NF) to study the synergistic interplay between the dual-elements codoping.

**Fig. 3:**
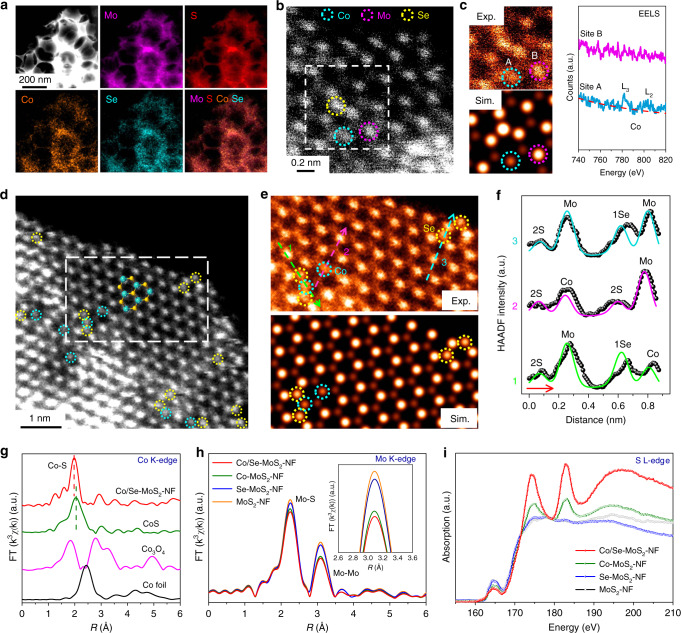
Structural and electronic properties of the Co/Se-doped MoS_2_ nanofoam (Co/Se-MoS_2_-NF). **a** HAADF-STEM image and corresponding EDX mappings of the Co/Se-MoS_2_-NF. **b** Atomic-resolution HAADF-STEM image of the Co/Se-MoS_2_-NF. **c** Enlarged and simulated images of the white dashed line region in **b** and the EELS spectra obtained at the site A and B via line scan. **d** Low pass filtered large area atomic-resolution HAADF-STEM image of the Co/Se-MoS_2_-NF. **e** Enlarged and simulated images of the white dashed line in **d**. **f** The measured intensity profile along three lines labeled in **e**. **g** Co K-edge k^3^-weighted EXAFS spectra of Co/Se-MoS_2_-NF sample in comparison with CoS, Co_3_O_4_, and Co foil, respectively. Mo K-edge k^3^-weighted EXAFS spectra (**h**) and S L-edge total electron yield X-ray absorption spectra (**i**) of Co/Se-MoS_2_-NF sample compared with Co-MoS_2_-NF, Se-MoS_2_-NF, and MoS_2_-NF. The Co/Se-MoS_2_-NF sample with 10.4% Co-doping content in atomic percentage was employed in these series of characterizations.

The TEM (Supplementary Fig. 10) and SEM (Supplementary Fig. 11) images show that the Co/Se-MoS_2_-NF possesses a similar morphology with that of the Se-MoS_2_-NF, containing assemble of small-sized MoS_2_ nano-flakes with abundant edges as presented in the HRTEM images (Supplementary Fig. 12). No nanoparticles or large clusters were observed in the TEM and HRTEM images of the Co/Se-MoS_2_-NF. The HAADF-STEM and corresponding EDX mappings (Fig. 3a) demonstrate the well-dispersion of Co and Se atoms in the structure. This is also confirmed by the XRD analysis showing no other diffraction peaks of Co- or Se-related crystal phases until the Co-doping content reaches 12.2% or more (Supplementary Fig. 13). In addition, the Co K-edge EXAFS characterizations indicate that the Co atoms in the Co/Se-MoS_2_-NF are mainly coordinated by S atoms and the Co–S bond in Co/Se-MoS_2_-NF is shorter compared with that of the standard CoS sample (Fig. 3g). A Co–Se bond (2.54 Å) is notably longer than a Co–S bond (2.41 Å) according to our DFT calculations but is not observed, which indicates that CoSe phase should not be formed. Besides, the Co valence state in the Co/Se-MoS_2_-NF is totally different from those of the Co foil, Co_3_O_4_, and CoS standard samples and falls in between those of the CoS and Co_3_O_4_ standard samples (Fig. 3g and Supplementary Fig. 14). Combined with the EXAFS results, it demonstrates that the Co atoms are covalently confined into the MoS_2_ lattice rather than being adsorbed on the surface or forming non-MoS_2_ hetero-phases such as CoS, CoSe, and Co_3_O_4_. Atomic-resolution HAADF-STEM imaging of a monolayer Co/Se-MoS_2_-NF shows the embedment of Co atoms with a lower contrast at the Mo site and Se atoms being obviously brighter at the S site (Fig. 3b), as confirmed by simulated HAADF-STEM image in Fig. 3c. The Co atom is further identified by using atomic-resolution electron energy loss spectroscopy (EELS), which shows the feature peaks of L_2_ and L_3_ edges of the Co atom at the site A with lower contrast (Fig. 3c). Distribution of Co and Se atoms being adjacent or separated to each other were observed in the HAADF-STEM image according to the contrast analysis (Fig. 3d), and were confirmed by the simulated HAADF-STEM images and the line intensity profiles (Fig. 3e, f).

Compared with pure Se-doping which can reduce the Mo–Mo coordination and increase the edge formation, Co/Se-codoping can further decrease both the Mo–Mo and Mo–S coordination as shown in the Mo K-edge EXAFS spectra (Figs. 1e, 3h). This is also reflected by the increasing peaks of bridging disulfide S_2_^2−^ in the S 2*p* XPS spectra of the Co/Se-MoS_2_-NF compared with those of Se-MoS_2_-NF and MoS_2_-NF (Supplementary Fig. 15), thus demonstrating the effect of the Co/Se-codoping in further promoting the formation of edges. Co/Se-codoping also leads to significant changes to the electronic structures of the S atoms. A notably negative shift in S 2*p* binding energies of the XPS was observed after the Co/Se codoping (Supplementary Fig. 16). In addition, the S L-edge XANES characterization shows that the intensity of the white line grows obviously higher after the Co/Se codoping compared with that of single-element Co- or Se-doped MoS_2_ with similar morphology (Fig. 3i). Co and Se atoms possess lower electronegativities than those of Mo and S atoms, so that the S atoms adjacent to the Co and Se dopants are more enriched with electrons, which synergistically modifies the electronic structures and hence the reactivity of S atoms^39,40^.

### HER performance of the Co/Se-MoS_2_-NF

**Fig. 4:**
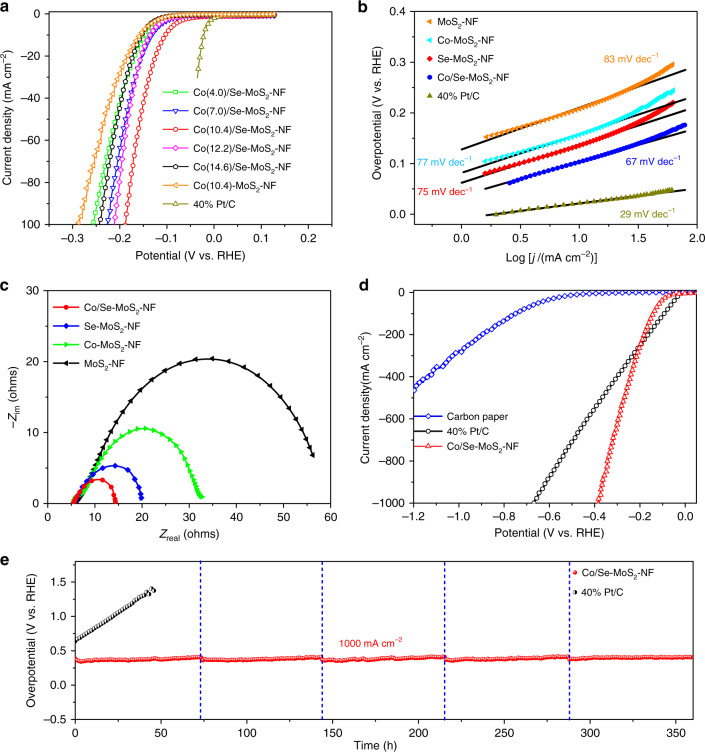
Synergetic effect of Co/Se-codoping on the HER performance of MoS_2_ nanofoam. **a** HER polarization curves for Co/Se-MoS_2_-NF with different Co-doping contents in comparison with Co-MoS_2_-NF and 40% Pt/C. All samples are drop-casted on glassy carbon electrode for measurements. The Tafel plots (**b**) and the electrochemical impedance spectroscopy (EIS) Nyquist plots (**c**) of Co/Se-MoS_2_-NF sample compared with Co-MoS_2_-NF, Se-MoS_2_-NF, MoS_2_-NF, and commercial 40% Pt/C. **d** HER polarization curves for Co/Se-MoS_2_-NF under large current densities in comparison with 40% Pt/C and carbon paper. Co/Se-MoS_2_-NF and 40% Pt/C are drop-casted on carbon paper for measurements. **e** The chronopotentiometric measurements of long-term stability for Co/Se-MoS_2_-NF and 40% Pt/C at 1000 mA cm^−2^. The vertical dotted lines in **e** mark the time span (every 72 h) of replacing the electrolyte in the 360 h chronoamperometry test. All the HER measurements were conducted in a 0.5 M H_2_SO_4_ electrolyte at 25 °C.

Considering the optimum Se-doping content of 9.1% in the Se-MoS_2_-NF offering the lowest overpotential, we adopted the same weight ratio of precursors for the Se and MoS_2_ and prepared a series of Co/Se-MoS_2_-NF samples with varied Co content from 4.0% to 14.6% to investigate the synergistic effect of Co/Se-codoping in tuning the HER activity. The onset overpotential of Co(4.0)/Se-MoS_2_-NF is significantly decreased compared with those of Se(9.1)-MoS_2_-NF and MoS_2_-NF (Fig. 4a). As the content of Co increases, the Co(10.4)/Se-MoS_2_-NF sample presents the lowest overpotentials of 104, 157, and 188 mV under the current densities of 10, 50, and 100 mA cm^−2^, respectively, which is superior to those of Co-doped MoSe_2_ and MoS_2_ catalysts (Supplementary Fig. 17 and Table 3). Then the activity decreases as the Co content further grows to 12.2% or larger, which may be due to the formation of other crystal phases at high Co content^21^.The Co(10.4)/Se-MoS_2_-NF sample also presents a much lower Tafel slope of 67 mV dec^−1^ than those of 77 mV dec^−1^ for the Co(10.4)-MoS_2_-NF, 75 mV dec^−1^ for the Se(9.1)-MoS_2_-NF and 83 mV dec^−1^ for the undoped MoS_2_-NF, respectively (Fig. 4b). This clearly demonstrates the superior HER kinetics over the Co/Se-codoped MoS_2_ via the Volmer–Heyrovsky mechanism, which differs from that over the Pt/C (40 wt.%) electrocatalyst via the Volmer–Tafel mechanism with a Tafel slope of 29 mV dec^−1^^41,42^.

The EIS measurements at a constant potential of 150 mV vs. reversible hydrogen electrode (RHE) indicate that the Co(10.4)/Se-MoS_2_-NF possesses the lowest *R*_ct_ value comparing with all other samples (Fig. 4c and Supplementary Table 2). The Tafel slope derived from the EIS reflects purely the charge-transfer kinetics of the catalytic process and is different from that derived from the linear sweep voltammetry (LSV) curves, where the difference stems from the electronic resistances of the catalysts^43,44^. The much similar values of the EIS and LSV Tafel slopes for the Co(10.4)/Se-MoS_2_-NF sample indicates an improved conductivity of the catalyst (Supplementary Fig. 18). The double-layer capacitance (*C*_dl_) extracted from the cyclic voltammetry (CV) measurements was used to estimate the electrochemically active surface areas. The Co(10.4)/Se-MoS_2_-NF shows a higher *C*_dl_ of 45.7 mF cm^−2^ compared with those of 26.3 mF cm^−2^ for the Co(10.4)-MoS_2_-NF, 30.7 mF cm^−2^ for the Se(9.1)-MoS_2_-NF, and 15.8 mF cm^−2^ for the MoS_2_-NF, respectively (Supplementary Figs. 19 and 20). This demonstrates that the Co(10.4)/Se-MoS_2_-NF possesses a higher catalytically active surface area and exposes more active sites^42^.

More interestingly, the Co(10.4)/Se-MoS_2_-NF exhibits both superior HER activities and stability at practically high current densities. At 208 mA cm^−2^, the overpotential becomes lower than that of the 40 wt.% Pt/C catalyst (Fig. 4d and Supplementary Fig. 21). At a higher large current density of 1000 mA cm^−2^, the overpotential is only 382 mV and significantly lower than that of 671 mV over the Pt/C catalyst. The faradic efficiency for H_2_ production in a wide range of working potential is well maintained at ~100%, which indicate that there is almost no other reduction product (Supplementary Fig. 22). To the best of our knowledge, such HER activity of the catalyst also surpasses those of all previously reported heteroatom-doped MoS_2_ catalysts (Supplementary Table 3). More importantly, the activity can be well maintained for more than 360 h without decay at the large current density of 1000 mA cm^−2^, while the activity of Pt/C catalyst will drop quickly within 50 h at the same test condition (Fig. 4e). The XRD (Supplementary Fig. 23) and SEM image (Supplementary Fig. 24) of the Co(10.4)/Se-MoS_2_-NF show no change in the crystallographic and morphology structure after the 360 h of durability test. The inductively coupled plasma optical emission spectrometry (ICP-OES) measurement shows that the content of Pt in the sample after the durability test is below the detection limit, thus indicating that Pt is not likely deposited on the Co(10.4)/Se-MoS_2_-NF sample. These results demonstrate that the dual-element Co/Se-codoping can synergistically enhance both the HER activity and stability of MoS_2_, even under industrial-level large current densities.

### Understanding the synergistic effect of Co/Se-codoping

**Fig. 5:**
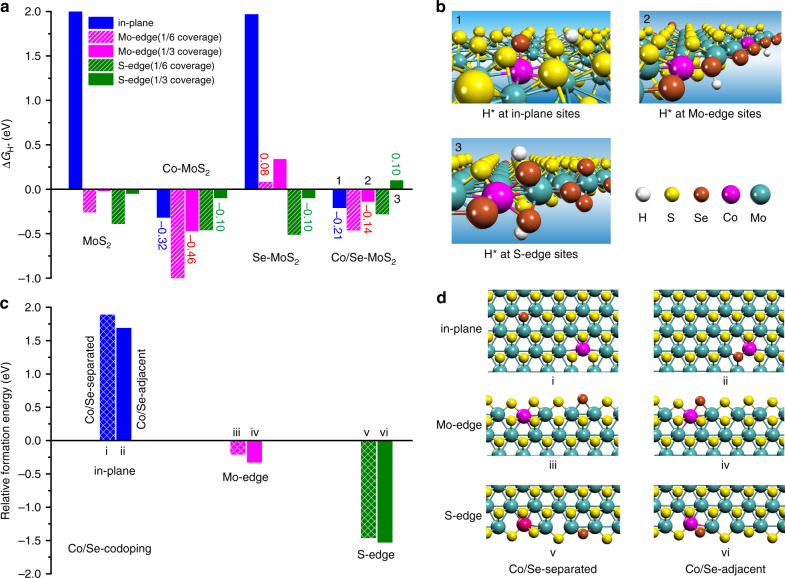
Theoretical studies of the Co-doping, Se-doping, and Co/Se-codoping effect in tuning the HER performance of MoS_2_. **a** The adsorption free energies of H* (ΔG_H*_) at the in-plane, Mo-edge, and S-edge sites of pure, Co-doped, Se-doped, and Co/Se-codoped MoS_2_, denoted as MoS_2_, Co-MoS_2_, Se-MoS_2_, Co/Se-MoS_2_, respectively. The hydrogen coverage at the Mo-edge is defined according to the terminating S or Se monomer sites, while that at the S-edge is defined according to the terminating S or Se dimer sites which are the active sites for the HER. **b** Adsorption structures of H* at the in-plane, Mo-edge and S-edge sites of Co/Se-MoS_2_. **c** Relative formation energies of the Co/Se-codoped structures with different doping configurations of Co and Se being separated (Co/Se-separated, grid bars) or adjacent (Co/Se-adjacent, solid bars) to each other. **d** Structures of Co/Se-codoped basal plane, Mo-edge, and S-edge with Co/Se-separated and Co/Se-adjacent configurations (Supplementary Fig. 26).

DFT calculations were performed to study the synergistic effect of dual-element Co/Se-codoping in improving the HER performance of MoS_2_ (Fig. 5). Co-doping significantly enhances the adsorption of hydrogen on the basal plane S site with adsorption free energy of H* (Δ*G*_H*_) decreasing from 2.01 to −0.32 eV (Fig. 5a). However, it also inevitably strengthens the adsorption of hydrogen on the Mo-edge S site with Δ*G*_H*_ of −0.47 eV, which leads to overbinding of H* and is unfavorable for the HER at the Co-doped Mo-edge sites. Introducing Se into the Co-doped MoS_2_ compensates the over-strong adsorption of H* on both the Co-doped in-plane and edge sites and thereby moderately weakens their adsorption activity for H*. At the Co/Se-codoped in-plane, Mo-edge, and S-edge sites, the Δ*G*_H*_ are increased to −0.21, −0.14, and 0.10 eV, respectively, from those of −0.32, −0.46, and −0.10 eV at the corresponding Co-doped sites (Fig. 5a, b). This indicates an improved HER activity at the Co/Se-codoped in-plane and Mo-edge sites with reduced overpotentials. In comparison, pure Se-doping only notably weakens the H* adsorption at the Mo-edge but still lead to a good HER activity at the Mo-edge and S-edge sites with Δ*G*_H*_ of 0.08 eV and −0.10 eV, respectively (Fig. 5a). Thus, the Co/Se-codoping brings a compromise between the enhancement effect of Co-doping and weakening effect of Se-doping on the hydrogen adsorption at the codoped sites, leading to more favorable hydrogen adsorption activities for the HER. In addition, Se-doping also prominently stabilizes the Co-doped basal plane and edges by forming Co–Se bonds (Fig. 5c, d), where the formation energies of Co/Se-codoped basal plane, Mo-edge, and S-edge can be reduced by 0.21, 0.12, and 0.07 eV per Co–Se bond formed, respectively, compared with those with Co and Se separated from each other. These results demonstrate a synergy between the activating effect of inner-layer Co-doping and the stabilization effect of surface Se-doping in the Co/Se-codoped MoS_2_, which significantly improves the HER performance by simultaneously enriching active sites and optimizing their hydrogen adsorption activities.

## Discussion

In summary, via co-confining Se in the surface and Co in the inner layer of the MoS_2_ combined with fabricating a nanofoam architecture, we achieve both ultrahigh activity and stability for the acidic HER. The activating effect of the inner-layer Co-doping combined with the stabilizing effect of the surface Se-doping enables the formation of abundant active sites in both the basal plane and the edges with optimized hydrogen adsorption activity at the codoped sites. The Co/Se-MoS_2_-NF presents the highest HER activity among the previously reported heteroatom-doped MoS_2_ catalysts under industrial-level large current densities. At a high current density of 1000 mA cm^−2^, the Co/Se-MoS_2_-NF exhibits a much lower overpotential of 382 mV than that of 671 mV over the 40 wt.% Pt/C catalyst, and the high activity can be maintained with long-term stability of more than 360 h without decay. Such a strategy of engineering the HER activity of MoS_2_ via co-confining multielements offers a promising and feasible route of developing high-performance and low-cost MoS_2_ catalysts for large-scale production of clean energy hydrogen.

## Methods

### Synthesis of SiO_2_ template

The uniform SiO_2_ nanosphere template was prepared by adding 9.6 g tetraethyl orthosilicate into 200 mL of ethanol solution under stirring for 10 min, followed by adding 8 mL of deionized water and 8 mL of ammonia. Then, the mixture was continuously stirred under room temperature until all the solvent was evaporated and was dried at 60 °C to obtain SiO_2_ template.

### Synthesis of Se-doped MoS_2_ nanofoam (Se-MoS_2_-NF)

The Se-MoS_2_-NF was synthesized through a one-pot chemical synthesis method. First, 0.4 g (NH_4_)_6_Mo_7_O_24_·4H_2_O, specified amount of selenium powder and 1.6 g SiO_2_ nanosphere powder were dispersed in 25 mL deionized water, followed by continuously stirring under room temperature until all the solvent was evaporated. Then, the gained solid was dried under 80 °C for 12 h. After that, the gained powder and 10 mL CS_2_ were both transferred into 40 mL stainless steel autoclave in the atmosphere of Ar and maintained at 400 °C for 4 h. The final product was treated with HF (aq.) under room temperature overnight, followed by washing several times with water and absolute ethanol and drying at 80 °C. For comparison, the MoS_2_ nanofoam (MoS_2_-NF) was synthesized via the same method as Se-MoS_2_-NF without adding selenium powder. The MoS_2_-FL was synthesized by using 0.9 g (NH_4_)_6_Mo_7_O_24_·4H_2_O dissolved in 20 mL deionized water and 10 mL CS_2_, and undergoing the same process as the synthesis of the MoS_2_-NF without using SiO_2_ template. All the doping contents in final samples were measured by ICP-OES. We have synthesized a series of Se-MoS_2_-NF samples with Se doping contents of 2.8, 5.7, 9.1, 10.3, and 12.9% in atomic percentage by the above methods.

### Synthesis of Co/Se-codoped MoS_2_ nanofoam (Co/Se-MoS_2_-NF)

For the synthesis of Co/Se-MoS_2_-NF samples, typically, 0.4 g (NH_4_)_6_Mo_7_O_24_·4H_2_O, 1.6 g SiO_2_ nanospheres powder, 30 mL deionized water, specified amount of Co(NO_3_)_2_·6H_2_O and selenium powder were mixed homogeneously, followed by continuously stirring under room temperature until the solvent was evaporated and drying at 80 °C. Then, the gained powder and 10 mL CS_2_ were transferred into 40 mL stainless steel autoclave and followed by the same process as the synthesis of the Se-MoS_2_-NF. For comparison, the Co-doped MoS_2_ nanofoam (Co-MoS_2_-NF) was synthesized by the same chemical method as the Co/Se-MoS_2_-NF without adding selenium powder. All the doping contents in final samples were measured by ICP-OES.

### Materials characterization

TEM, HAADF-STEM, HRTEM, and EDX mappings measurements were carried out on the Phillips Analytical FEI Tecnai20 electron microscope operated at an accelerating voltage of 200 kV. SEM measurements were carried out on Hitachi S5500 operated at 30 kV. Atomic-resolution HAADF-STEM images and EELS mapping were performed on a spherical aberration-corrected Nion U-HERMES100. The experiments were conducted at 60 kV accelerating voltage to reduce radiation damage and also with relatively low electron dose. EELS spectra processing was performed using the open-source software HYPERSPY (formerly EELSLAB) to remove X-ray strikes^45^. Atomic-resolution HAADF-STEM image simulation was performed in the QSTEM software. XRD patterns were recorded on Rigaku Ultima IV diffractometer. Cu Kα radiation (*γ* = 0.15406 mm) at 40 kV and 30 mA was used as the X-ray source. XPS measurements were carried out on a Thermo ESCALAB 250Xi spectroscope using Al Kα x-rays as the excitation source. Raman measurements were performed on a home-modified Invia confocal Raman microscope (Renishaw, UK) with Leica DM2500 microscopes, which was operated with a 633 nm excitation laser at a power of 26.7 μW. XANES and EXAFS were measured at the BL14W1 beamline of the Shanghai Synchrotron Radiation Facility (SSRF) recorded in a transmission mode. The S L-edge XANES spectra were collected in total electron yield mode at the beamline BL10 in Hefei National Synchrotron Radiation Laboratory (NSRL). ICP-OES test was conducted on Shimadzu ICPS-8100.

### Electrochemical measurements

Electrochemical measurements were performed in a three-electrode electrochemical cell equipped with a gas flow controlling system on electrochemical workstations (CHI 760E and CHI 680 C). We employed the glassy carbon rotating disk electrode with a diameter of 5 mm covered by a thin catalyst film as working electrode to perform HER measurements at low-level current densities of 10, 50, and 100 mA cm^−2^. Typically, 4 mg catalyst and 2 mg carbon black (Vulcan XC-72) was suspended in 1 mL ethanol with 20 μL Nafion solution (5 wt.%, Du Pont) to form a homogeneous ink assisted by ultrasound. Then 25 μL of the ink was spread onto the surface of glassy carbon by a micropipette and dried under room temperature. The final loading amount of catalysts on work electrode is 0.5 mg cm^−2^. Graphite rod and Ag/AgCl (saturated KCl-filled) were used as counter electrode and reference electrode, respectively. HER tests were conducted in an Ar-saturated 0.5 M H_2_SO_4_ electrolyte at 25 °C under 1600 rpm. The LSV polarization curve was measure at 5 mV/s in the potential range of −0.1 to 1.0 V (vs. Ag/AgCl). Before measurements, the sample were repeatedly swept from −0.4 to 0.3 V (vs. Ag/AgCl) in the electrolyte until steady voltammogram curve was obtained. The long-term stability tests were performed by using chronopotentiometric measurement on the carbon fiber paper (CFP) with a catalyst loading of 0.5 mg cm^−2^. EIS was evaluated at −0.15 V (vs. RHE) between 100 kHz and 0.1 Hz with a 5 mV AC potential perturbation. Tafel analysis from EIS measurements was performed at various working potential from 100 kHz to 0.1 Hz with a 15 mV AC potential perturbation. To determine the double-layer capacitance (*C*_dl_), the CV were performed at various scan rates (20, 40, 60 mV s^−1^, etc.) between 0.1 V and 0.2 V (vs. RHE).

We loaded the catalyst on carbon paper to measure the HER activity and stability at large current densities up to 1000 mA cm^−2^. The Tafel slopes of the Pt/C catalyst loaded on carbon paper and glassy carbon electrode are almost the same, which indicating that their HER kinetics are identical (Supplementary Fig. 25). The Fig. 4d, e show polarization curves and chronopotentiometric measurements under large current densities, respectively, which were obtained by using a three-electrode H-type electrochemical cell equipped with a gas flow controlling system. For the preparation of the working electrode, 4 mg catalyst and 2 mg carbon black (Vulcan XC-72) were suspended in 0.5 mL ethanol with 25 μL Nafion solution (5 wt.%, Du Pont) to form a homogeneous ink assisted by ultrasound. Then, the ink was dropped on CFPs (1 cm × 1 cm) and dried under room temperature. For the characterization of HER polarization curves, an Ag/AgCl (saturated KCl-filled) electrode and graphite rod were used as reference and counter electrode, respectively. For the characterization of chronopotentiometric test, Pt mesh was used as the counter electrode owing to the instability of graphite rod under large current densities. In order to avoid effectively the possible Pt contamination during measurements, cathode and anode compartments were separated with Nafion 117 membrane in H-type electrochemical cell. To weaken the influence of reactant concentration for catalyst activity, the electrolyte was replaced every 72 h.

All potentials were 85% iR corrected and referenced to the RHE by the equation E(RHE) = E(Ag/AgCl, saturated KCl-filled)—iR + 0.227. The iR correction was performed according to a previously reported method^46,47^. The R for iR correction was determined by impedance measurements.

### Calculation of the faradaic efficiency

The controlled potential electrolysis was carried out to determine the faradaic efficiency of H_2_ using a three-electrode H-type electrochemical cell. During the controlled potential electrolysis, the electrolyte in both anodic and cathodic compartments were stirred slightly to enhance the diffusion of reactant. Quantification of the H_2_ were performed using the gas chromatograph (Shimadzu GC 2014) with a thermal conductivity detector. The faradaic efficiency of H_2_ are calculated from GC chromatogram peak areas at a given potential as follow:

1 $$i_{{\rm{H}_{2}}}\,=\,\upsilon _{{\rm{H}_{2}}}\,\times\,V\,\times\,\frac{{2Fp_0}}{{RT}},$$

2 $$FE_{\rm{H}_{2}}\,=\,\frac{{i_{\rm{H}_{2}}}}{{i_{\rm{total}}}}\,\times\,100{\mathrm{\% }},$$

where $i_{\rm{H}_{2}}$ is partial current density for H_2_, *i*_total_ is total current density, $\upsilon _{\rm{H}_{2}}$ is volume concentration of H_2_ based on the calibration of the GC, *V* is gas volume flow rate, and *F* is the Faraday’s constant 96,485 C mol^−1^. *p*_0_ = 101.325 kPa, *R* = 8.314 J mol^−1^ K^−1^, and *T* = 298.15 K.

### DFT calculations

DFT calculations were performed using the Vienna Ab-initio Simulation Package^48–51^. The projector augmented-wave pseudopotential method with Perdew–Burke–Ernzerhof exchange-correlation functional and a plane-wave cutoff energy of 400 eV was adopted^52–55^. The standard version of PAW pseudopotentials without using semi-core states were used for all elements. Van der Waals correction was calculated using the Zero-damping DFT-D3 method of Grimme^56,57^. A tri-layer model of MoS_2_ was built in a 6 × 6 supercell to simulate the in-plane doping of Co and Se. A nanoribbon model of MoS_2_ with six repeated units along the belt direction was built to simulated the Mo-edge and S-edge. The Mo edge was saturated with S monomers and the S edge was saturated with a combination of S monomers and dimers^58,59^. The vacuum thicknesses were set larger than 15 Å between the layers or the ribbons. A Monkhorst–Pack k-point sampling of 1 × 1 × 1 was selected for all models^60^. In structural optimizations, the residual forces between atoms were converged below 0.02 eV/Å. Dipole correction was applied to decouple the interactions between the slabs or nanoribbons.

The free energy of (H^+^ + e^−^) was calculated as that of ½ H_2_. The free energy of gas molecules was calculated as *E*_total_ + ZPE + ${\int}_0^T {C_p} dT$ − *TS*, where *E*_total_ is DFT calculated total energy, ZPE is the zero-point energy, *T* is temperature, ${\int}_0^{298.15} {C_p} dT$ is the integrated heat capacity from 0 K to *T*, and *S* is the entropy. For the ZPE + ${\int}_0^T {C_p} dT$ − *TS* parts, we adopt the experimental data from the NIST database (Supplementary Table 4). The free energies of adsorbed species were calculated as *E*_total_ + ZPE + ${\int}_0^T {C_{v(vib)}} dT$ − *TS*_vib_, where the ${\int}_0^T {C_{v(vib)}} dT$ and *S*_vib_ are the integrated heat capacity from 0 K to *T* and the entropy parts from non-imaginary vibrations based on harmonic oscillation approximation.

The formation energies were calculated by using the free energies of H_2_, H_2_S, pristine MoSe_2_, and MoS_2_, and Co bulk as the references. The chemical potential of each element is calculated as:

3 $$\mu \left({\rm{H}}\right)\,=\,1/2G\left( {\rm{H}_{2}} \right),$$

4 $$\mu \left({\rm{S}}\right)\,=\,G\left( {\rm{H}_{2}{\rm{S}}} \right)\,-\,G\left( {\rm{H}_{2}} \right),$$

5 $$\mu \left( {\rm{Mo}} \right)\,=\,G\left( {\rm{MoS}_{2}} \right)\,-\,2\mu \left( S \right),$$

6 $$\mu \left( {\rm{Se}} \right)\,=\,1/2\left[ {G\left( {\rm{MoSe}_{2}} \right)\,-\,\mu \left( {\rm{Mo}} \right)} \right],$$

7 $$\mu \left( {\rm{Co}} \right)\,=\,1/2G\left( {{\rm{Co}}\,-\,{\rm{bulk}}} \right) \ast.$$

*The supercell of hcp Co bulk contains two Co atoms.

Then the formation energy (*E*_form_) of a structure Mo_*x*_Co_*y*_S_*z*_Se_*m*_ is given by:

8 $$E_{\rm{form}}\,=\,E_{\rm{total}}\left( {{\rm{Mo}}_{\rm{x}}{\rm{Co}}_{\rm{y}}{\rm{S}}_{\rm{z}}{\rm{Se}}_{\rm{m}}} \right)\,-\,x\mu \left( {\rm{Mo}} \right)\,-\,y\mu \left( {\rm{Co}} \right)\,-\,z\mu \left({\rm{S}}\right)\,-\,m\mu \left( {\rm{Se}} \right).$$

The relative formation energy of a Co-doped, Se-doped, or Co/Se-codoped MoS_2_ edge is calculated by using its formation energy minus that of the corresponding undoped pure MoS_2_ edge. This is to decouple the doping effect on the formation energy from the that of creating undoped pure MoS_2_ edges.

## Supplementary information

Supplementary Information

Peer Review File

## Data Availability

The data that support the findings of this study are available from the corresponding authors on request.
