# Supplementary material for: Boosting hydrogen evolution on MoS2 via co-confining selenium in surface and cobalt in inner layer
Source: Nat Commun. 2020 Jul 3;11:3315. doi: 10.1038/s41467-020-17199-0 (PMC7334232; doi:10.1038/s41467-020-17199-0)
Supplement: Supplementary file 1 — Supplementary Information [file 41467_2020_17199_MOESM1_ESM.pdf]

Supplementary Information

Boosting hydrogen evolution on MoS_2 via co-confining selenium in surface and cobalt in inner layer

Zheng et al.

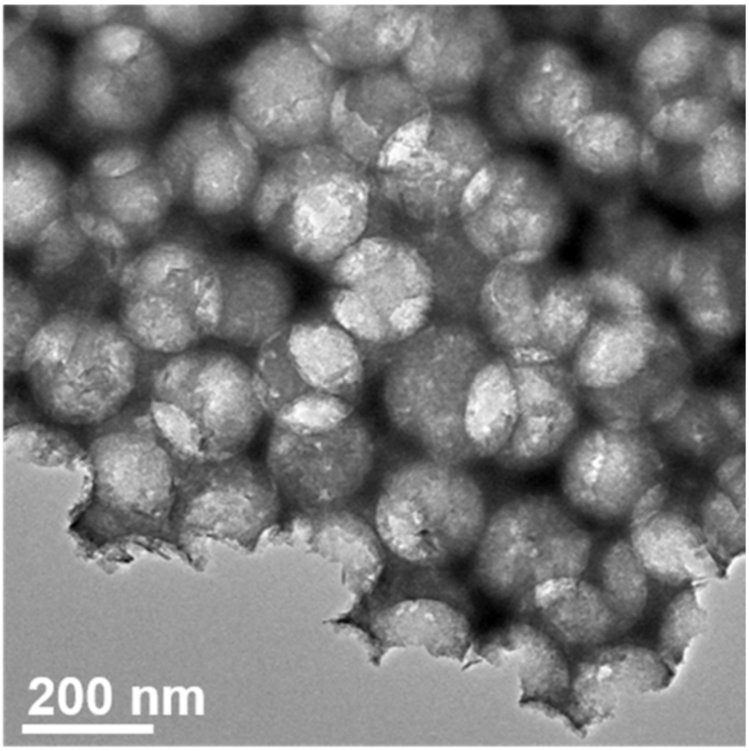

Supplementary Figure 1 | TEM image of the Se(9.1)-MoS_2-NF.

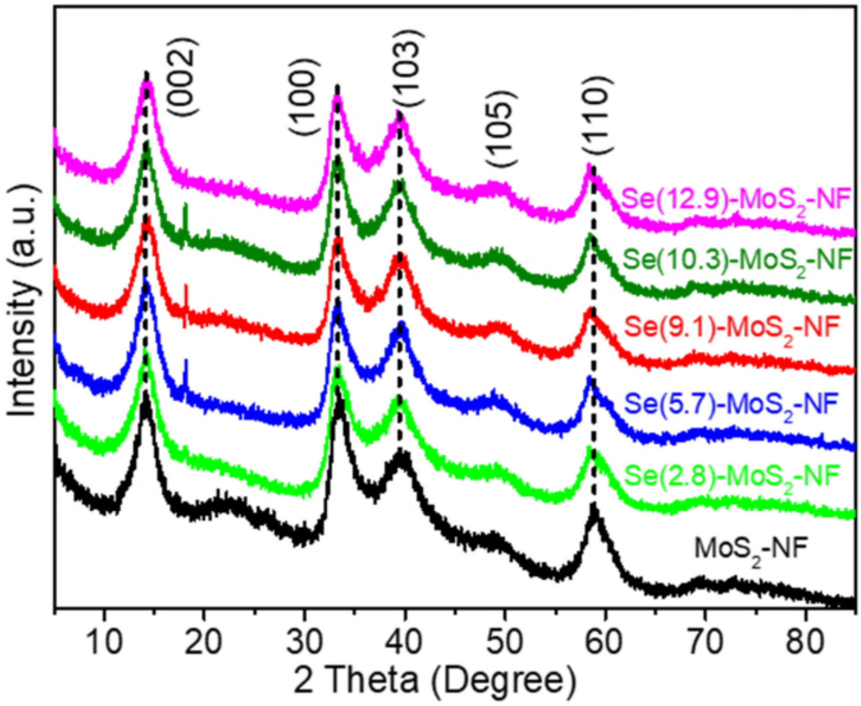

Supplementary Figure 2 | XRD patterns of a series of Se-MoS_2-NF samples in comparison with MoS_2-NF.

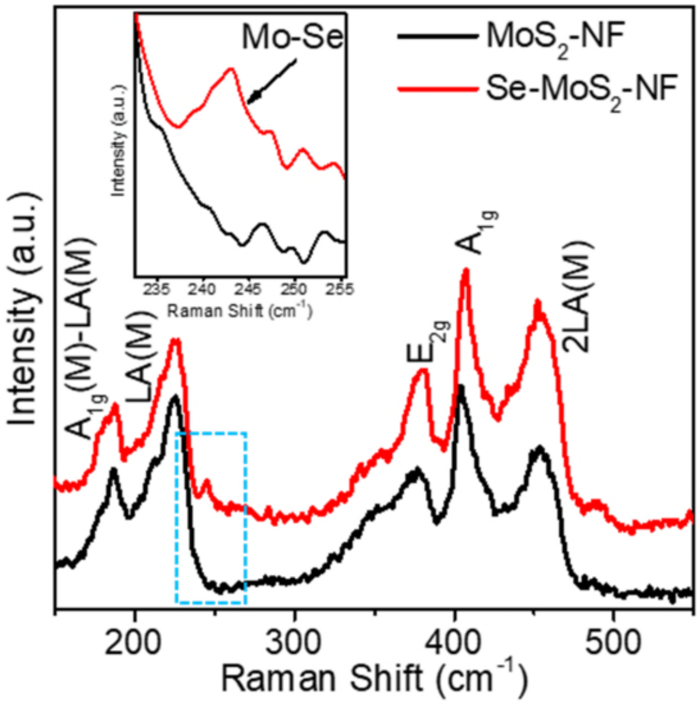

Supplementary Figure 3 | Raman spectra of the Se(9.1)-MoS_2-NF and MoS_2-NF.

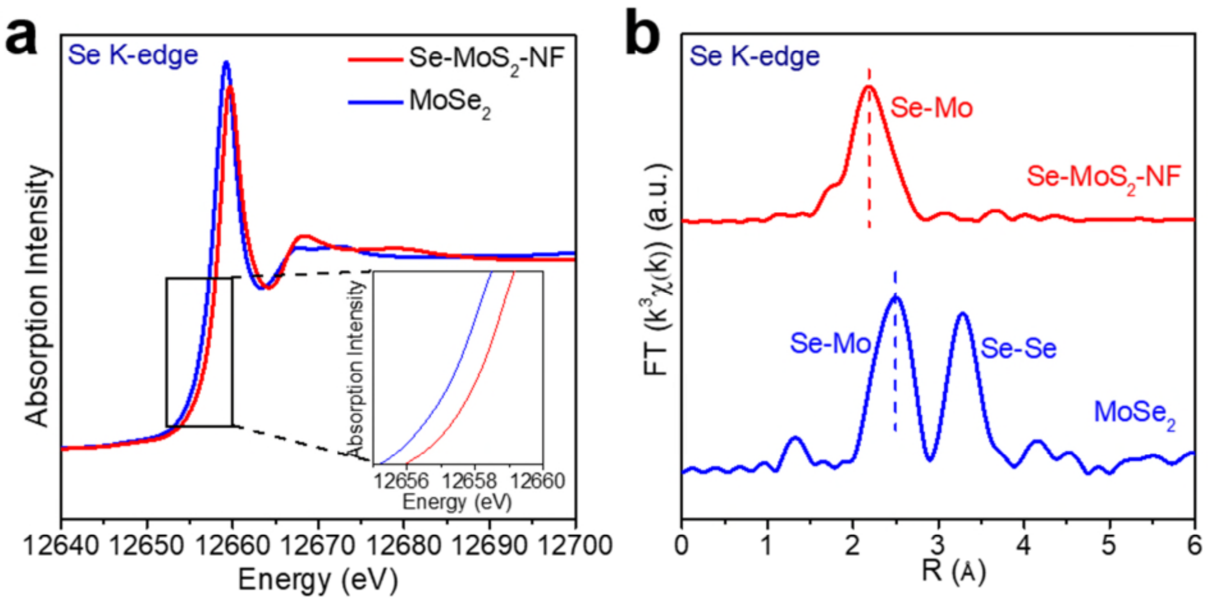

Supplementary Figure 4 | Comparison in the XAFS spectra of the Se K-edge of the Se-MoS_2-NF and $MoSe_2$ samples. a,**b**, The Se K-edge XANES spectra (**a**) and k^3-weighted EXAFS spectra (**b**) of the Se(9.1)-MoS_2-NF and $MoSe_2$.

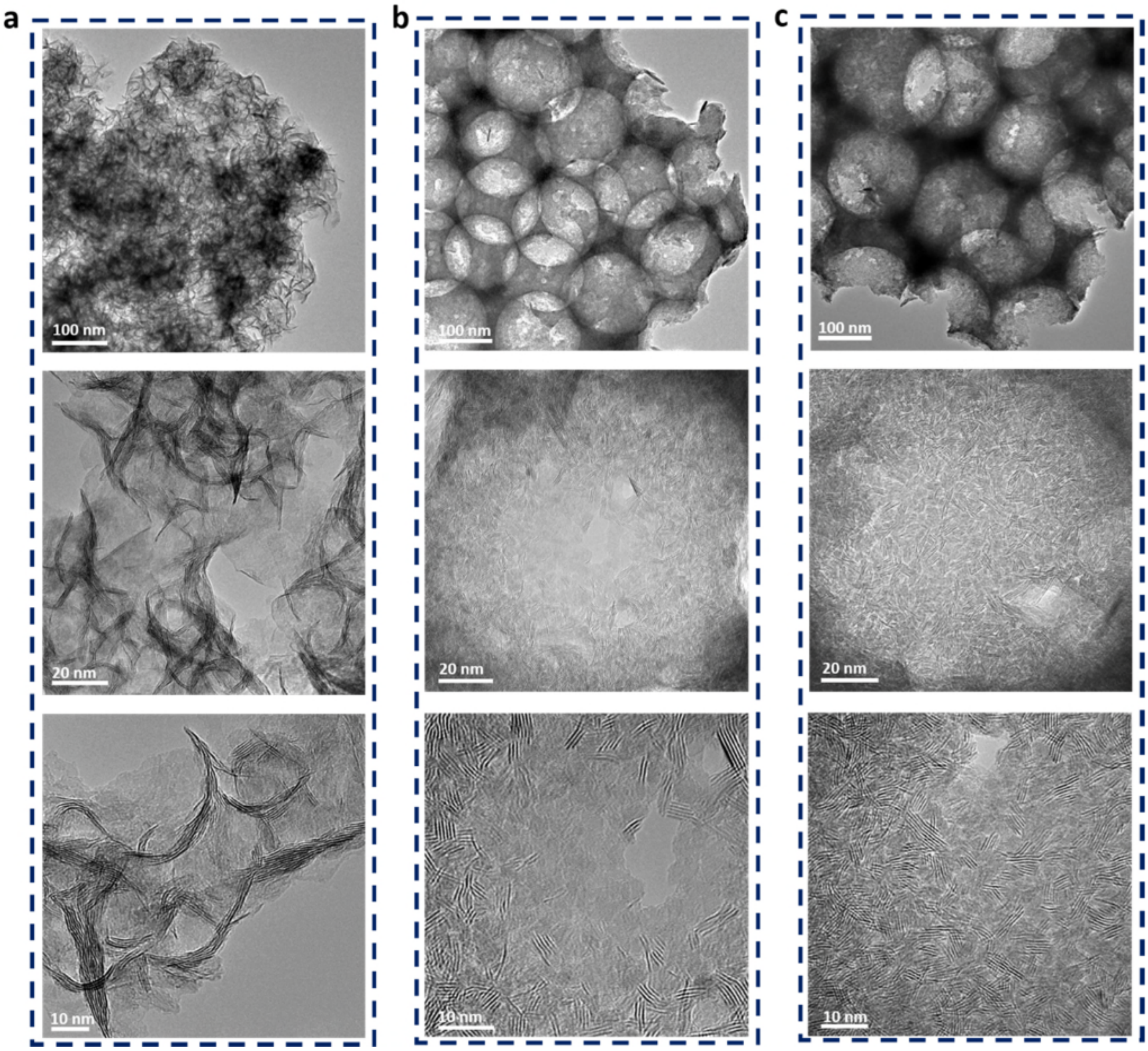

Supplementary Figure 5 | a-c, HRTEM images of the MoS_2-FL (**a**), MoS_2-NF (**b**) and Se(9.1)-MoS_2-NF (**c**).

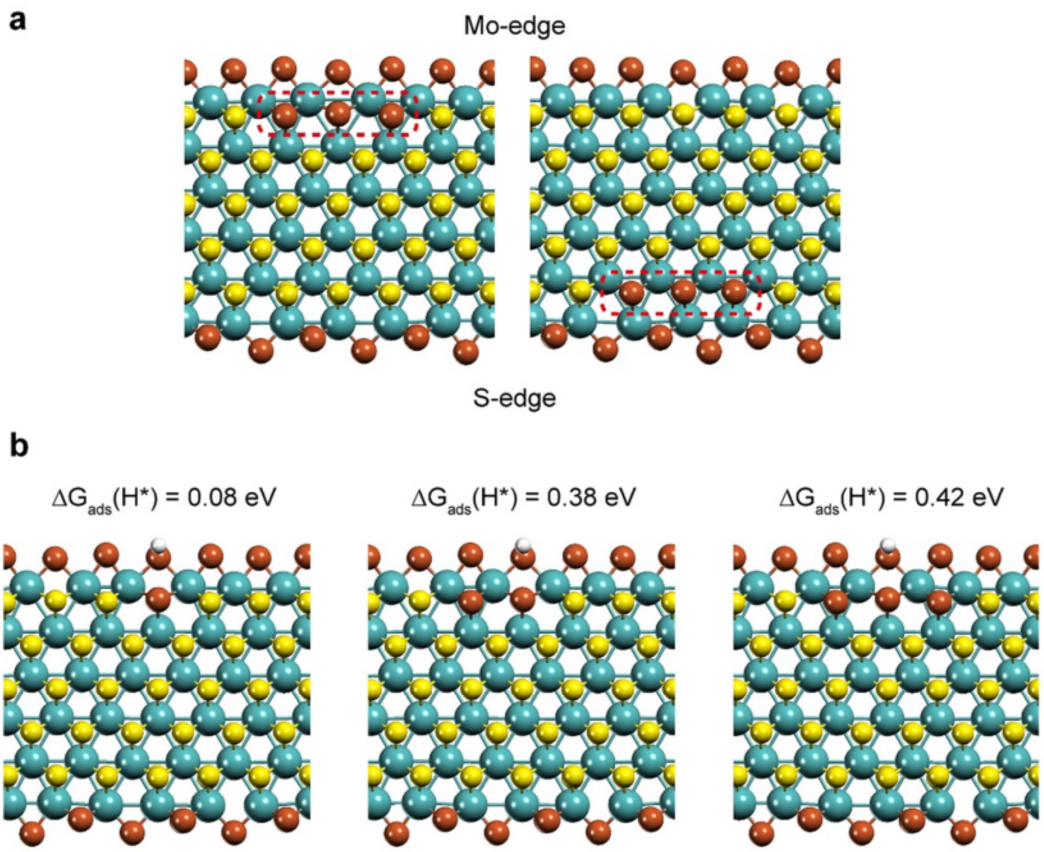

Supplementary Figure 6 | a, Nanoribbon models of MoS_2 for simulating the over-doping effect of Se on the edge reactivity. The red-dotted square labels the further substitution of S by Se at the Se-terminated Mo- or S-edges. **b,** Adsorption free energies of H* (ΔG_{ads}(H*)) at the Se site of the Se-terminated Mo-edge with increasing doping content of Se.

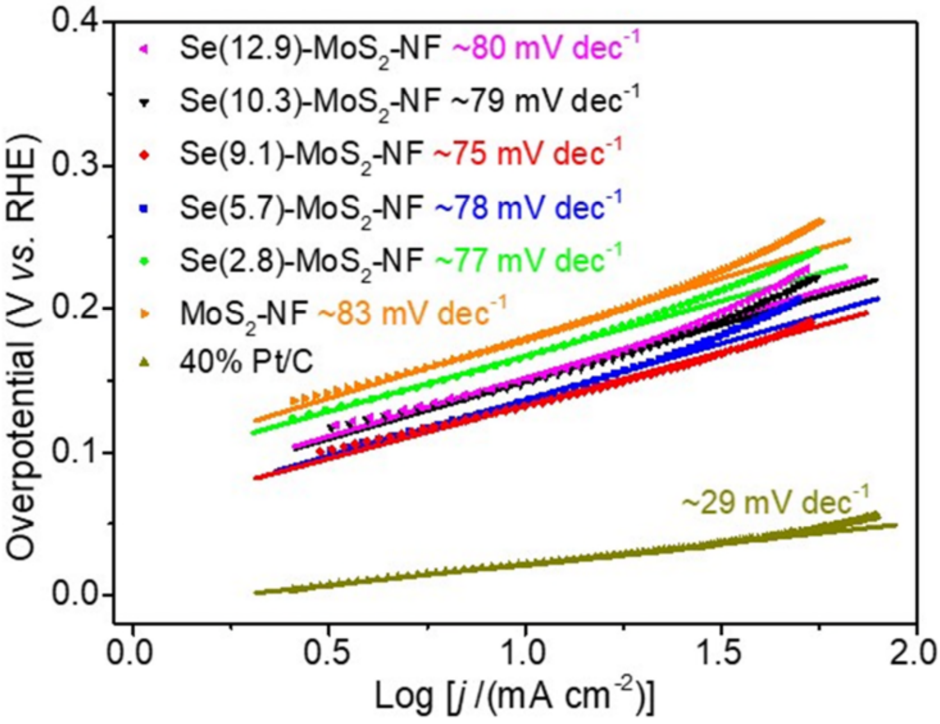

Supplementary Figure 7 | The Tafel slope plots of the Se-MoS_2-NF samples with different Se doping contents in comparison with those of the MoS_2-NF and 40% Pt/C.

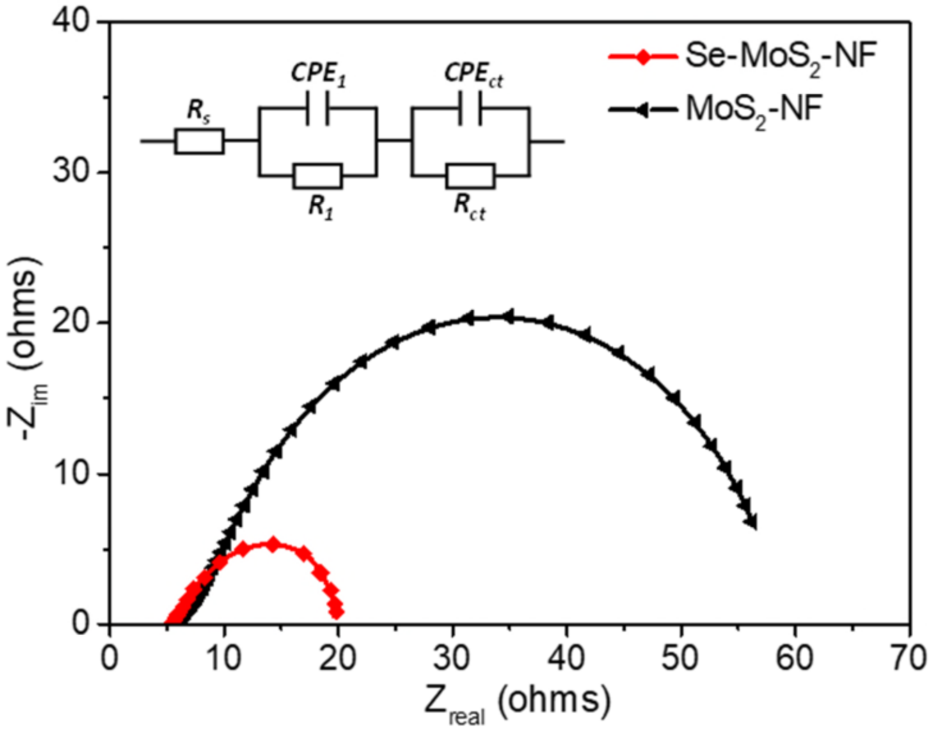

Supplementary Figure 8 | Electrochemical impedance spectroscopy (EIS) Nyquist plots of MoS_2-NF and Se(9.1)-MoS_2-NF at 150 mV versus RHE, the inset is the corresponding equivalent circuit model. The equivalent circuit contains a resistor (R_s), in series connection with two parallel units of a constant phase element (CPE_1, CPE_{ct}) and a resistor (R_1, R_{ct}), where R_s represents the solution resistance, the CPE_1-R_1 pair is probably related to the interfacial resistance resulting from the electron transport between the catalysts and GCE, and the CPE_{ct}-R_{ct} pair reflects the charge transfer resistance at the interface between catalysts and the electrolyte.

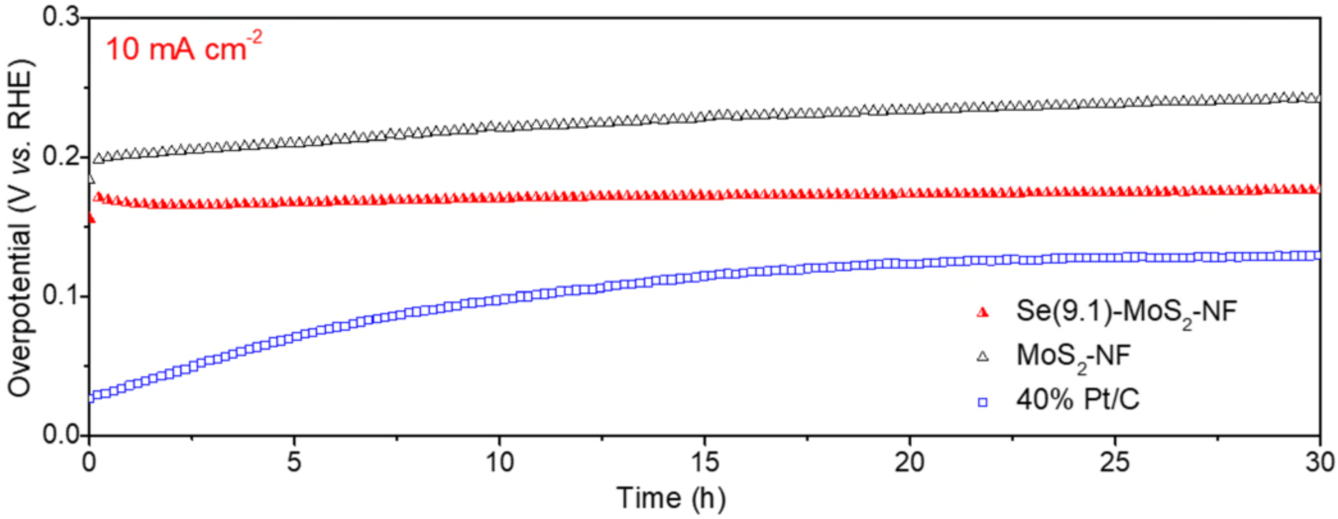

Supplementary Figure 9 | The chronopotentiometric measurements of long-term stability for the MoS_2-NF, Se(9.1)-MoS_2-NF and 40% Pt/C at 10 mA cm^{-2} for 30 hours.

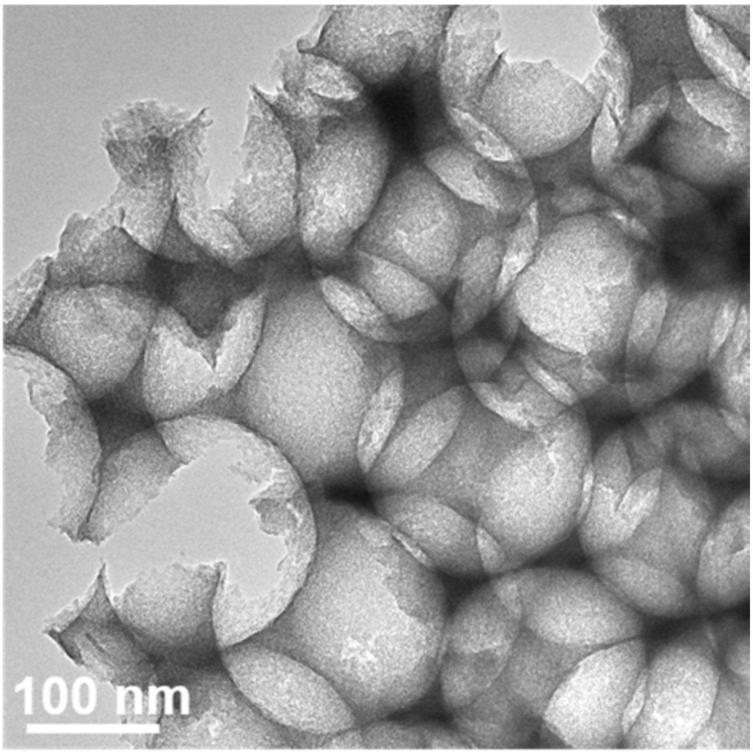

Supplementary Figure 10 | TEM image of the Co(10.4)/Se-MoS_2-NF.

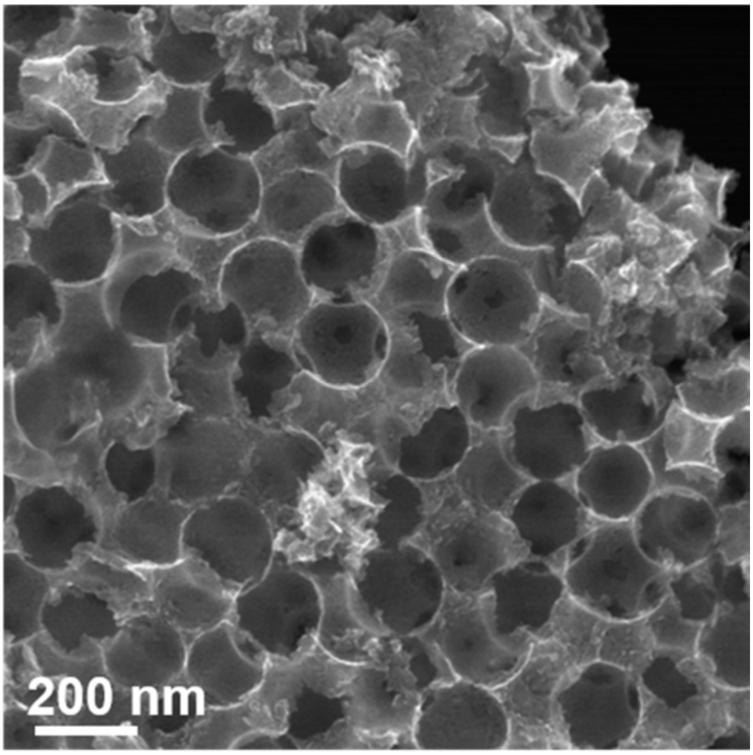

Supplementary Figure 11 | SEM image of the Co(10.4)/Se-MoS_2-NF.

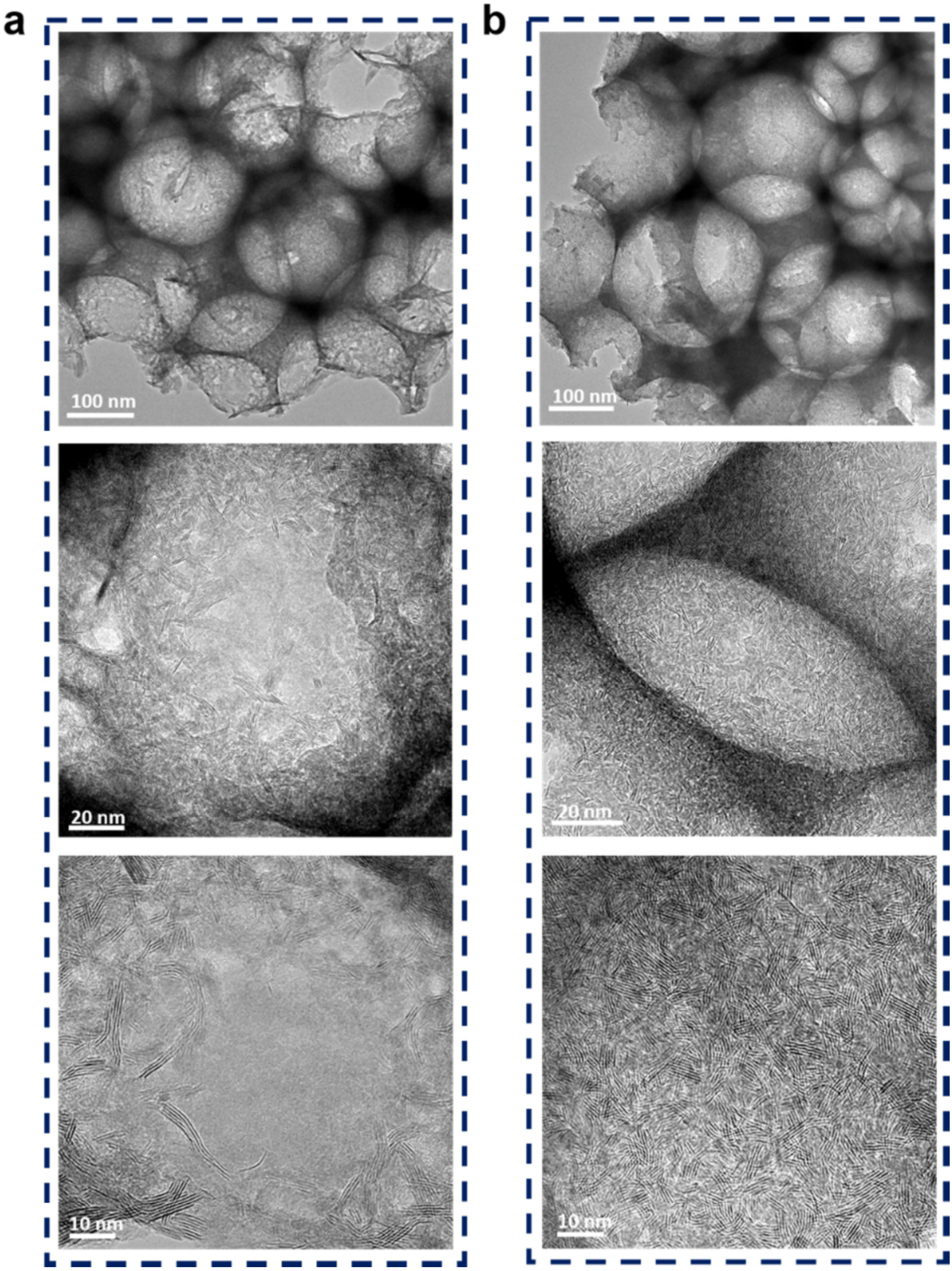

Supplementary Figure 12 | a,**b**, HRTEM images of the Co(10.4)-MoS_2-NF (**a**) and Co(10.4)/Se-MoS_2-NF (**b**).

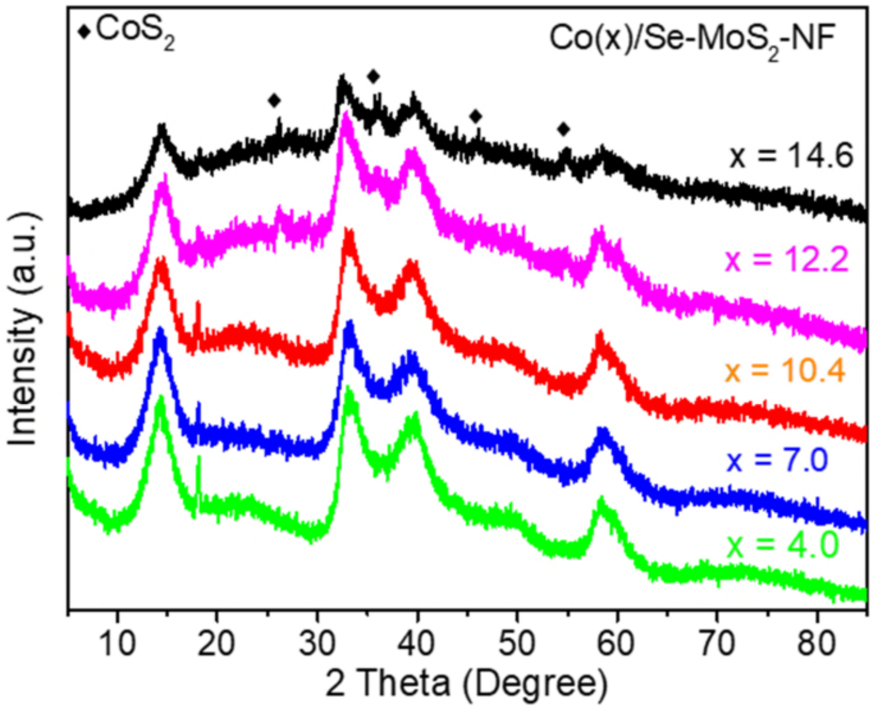

Supplementary Figure 13 | XRD patterns of a series of Co/Se-MoS_2-NF samples.

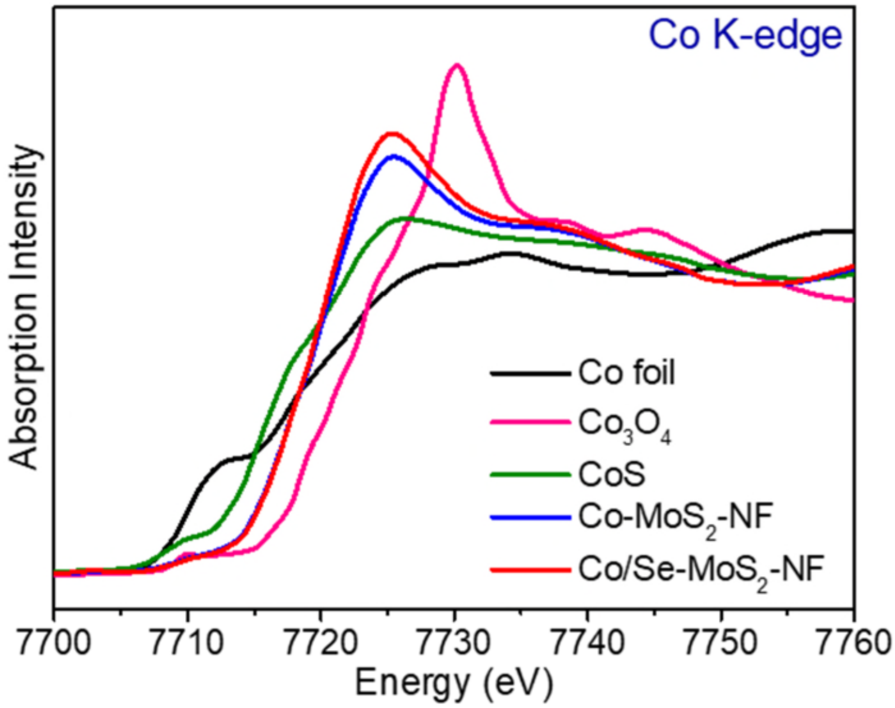

Supplementary Figure 14 | The Co K-edge XANES spectra of the Co(10.4)/Se-MoS_2-NF, Co(10.4)-MoS_2-NF, CoS, Co_3O_4 and Co foil.

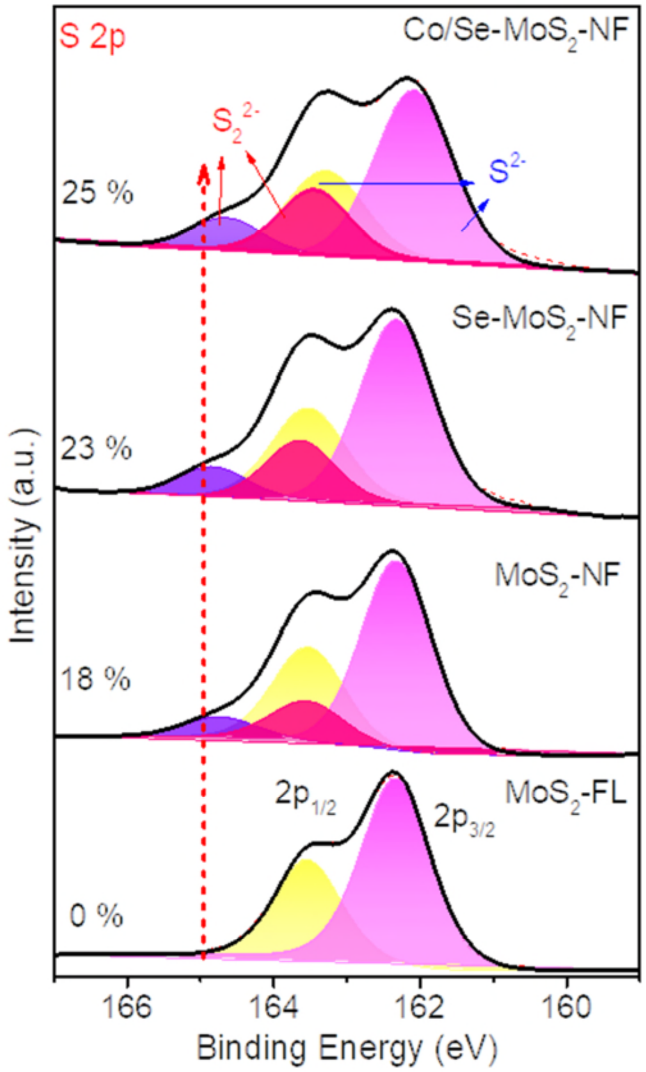

Supplementary Figure 15 | Decomposition of the S 2p XPS spectra of the Co(10.4)/Se-MoS_2-NF, Se(9.1)-MoS_2-NF, MoS_2-NF and MoS_2-FL catalysts.

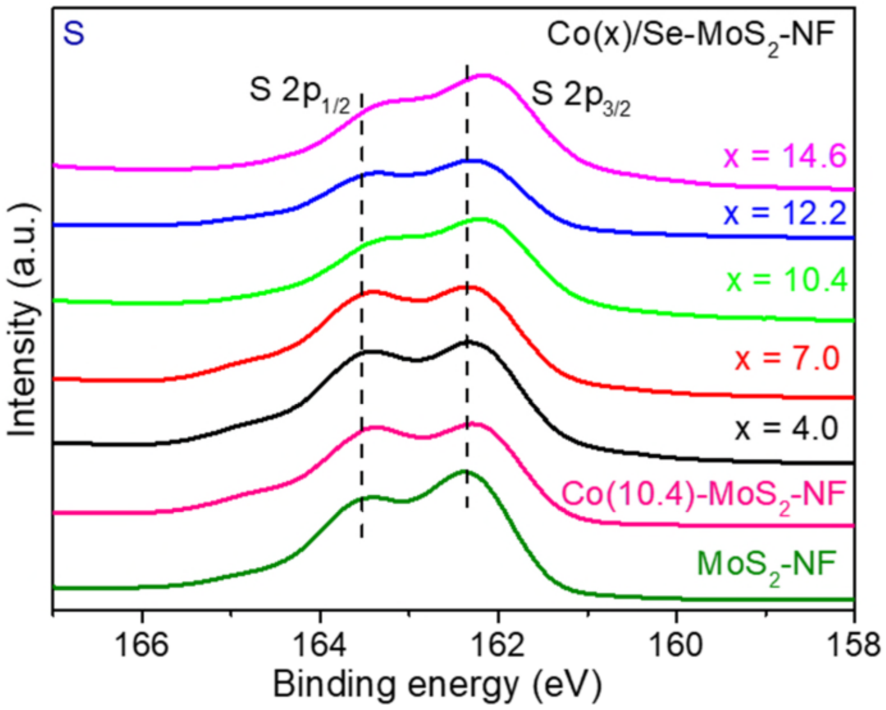

Supplementary Figure 16 | The S 2p XPS spectra of a series of Co/Se-MoS_2-NF in comparison to those of the Co(10.4)-MoS_2-NF and MoS_2-NF.

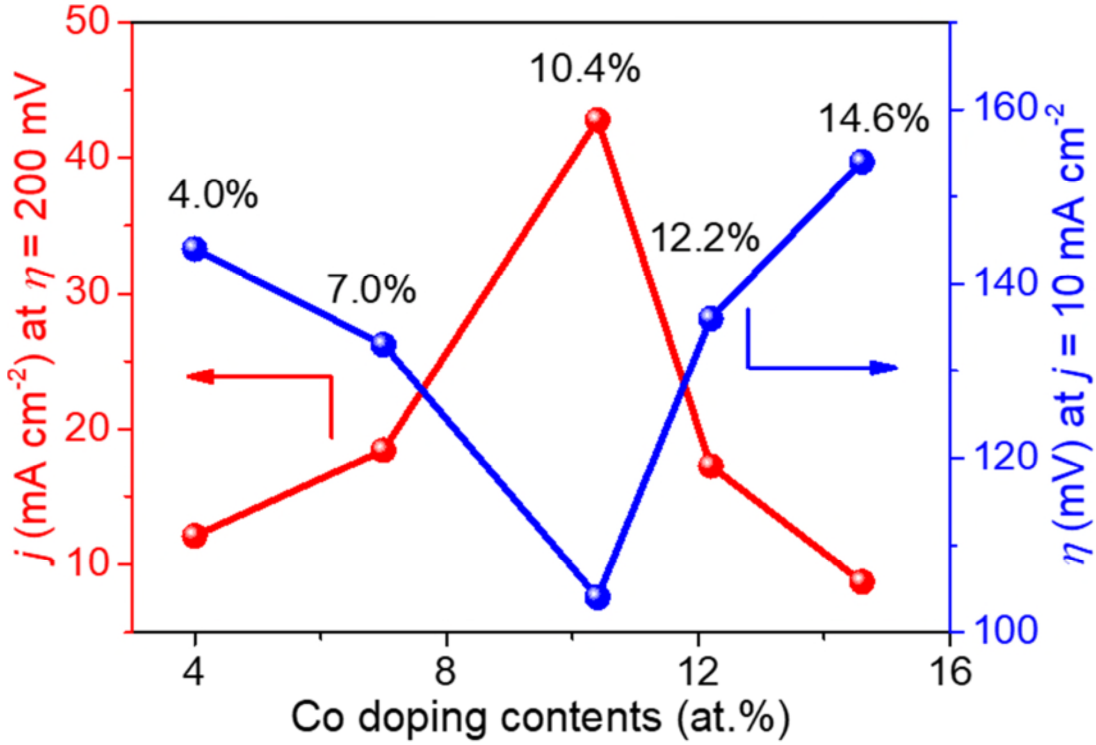

Supplementary Figure 17 | The HER activity of a series of Co(x)/Se-MoS_2-NF catalysts in dependence on the Co-doping content, measured at a constant overpotential of 200 mV (red) and a constant current density of 10 mA cm^{-2} (blue), respectively.

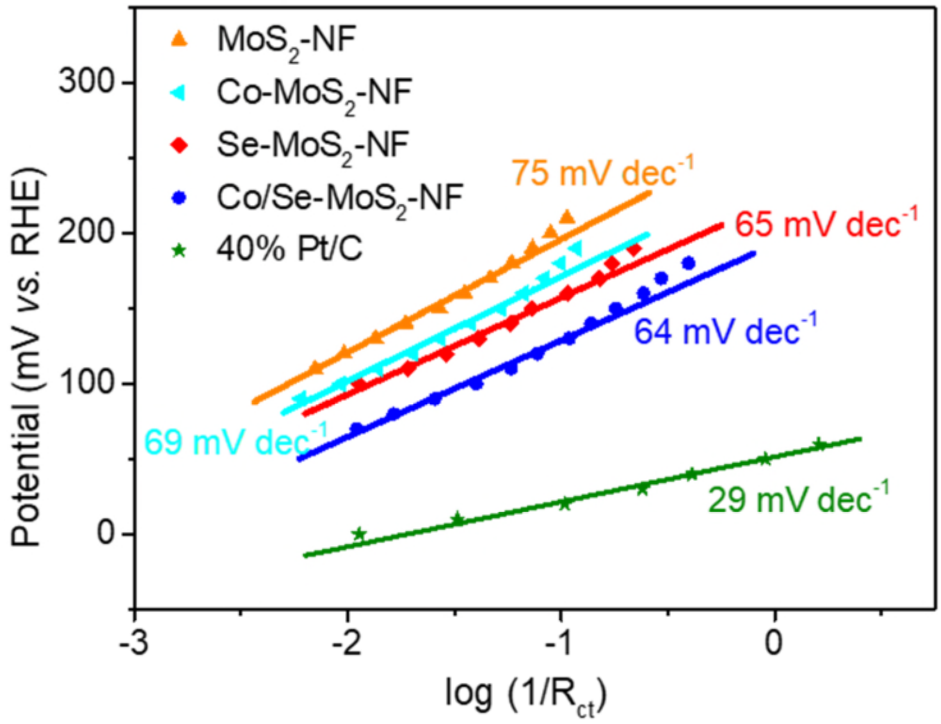

Supplementary Figure 18 | Analysis of Tafel slopes from the EIS measurements of the MoS_2-NF, Co(10.4)-MoS_2-NF, Se(9.1)-MoS_2-NF, Co(10.4)/Se-MoS_2-NF and commercial 40% Pt/C.

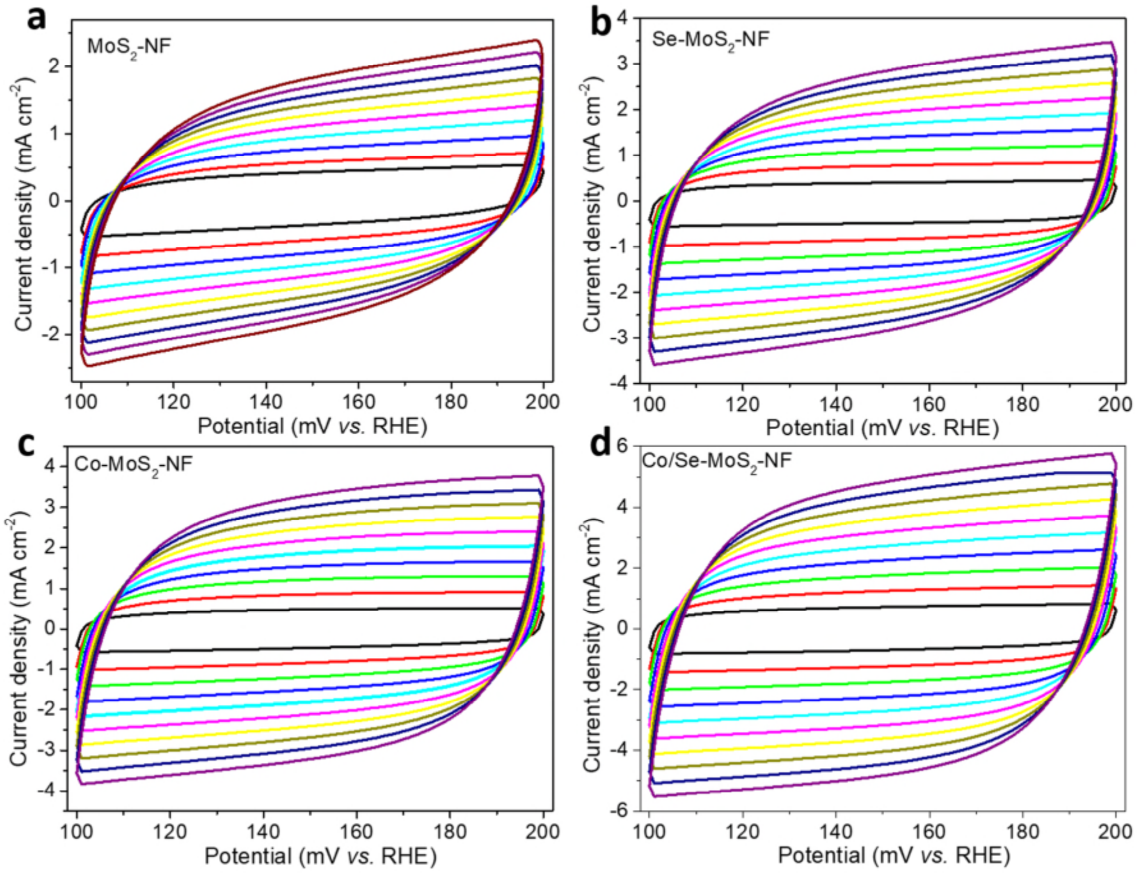

Supplementary Figure 19 | Cyclic voltammetry curves of the MoS_2-NF (**a**), Se(9.1)-MoS_2-NF (**b**), Co(10.4)-MoS_2-NF (**c**), and Co(10.4)/Se-MoS_2-NF (**d**), measured at various scan rates (20, 40, 60 mV s^{-1}, etc.) from 0.1 V to 0.2 V vs. RHE.

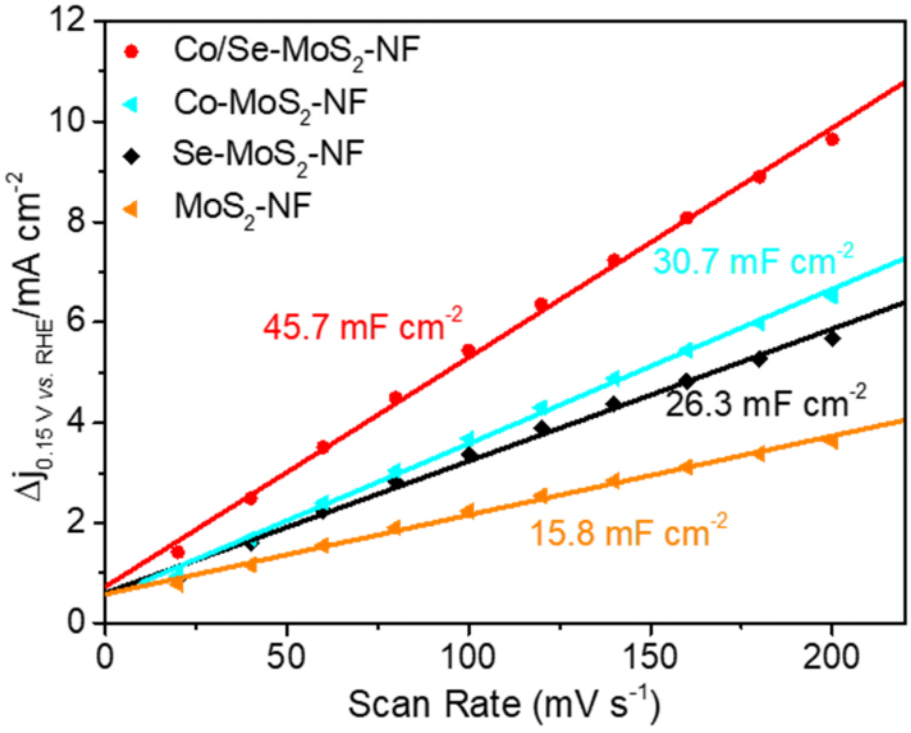

Supplementary Figure 20 | The differences in current density variation ($\Delta j = j_a - j_c$) at an overpotential of 150 mV (versus RHE) plotted against scan rate fitted to a linear regression, which can be used to estimate the C_{dl}.

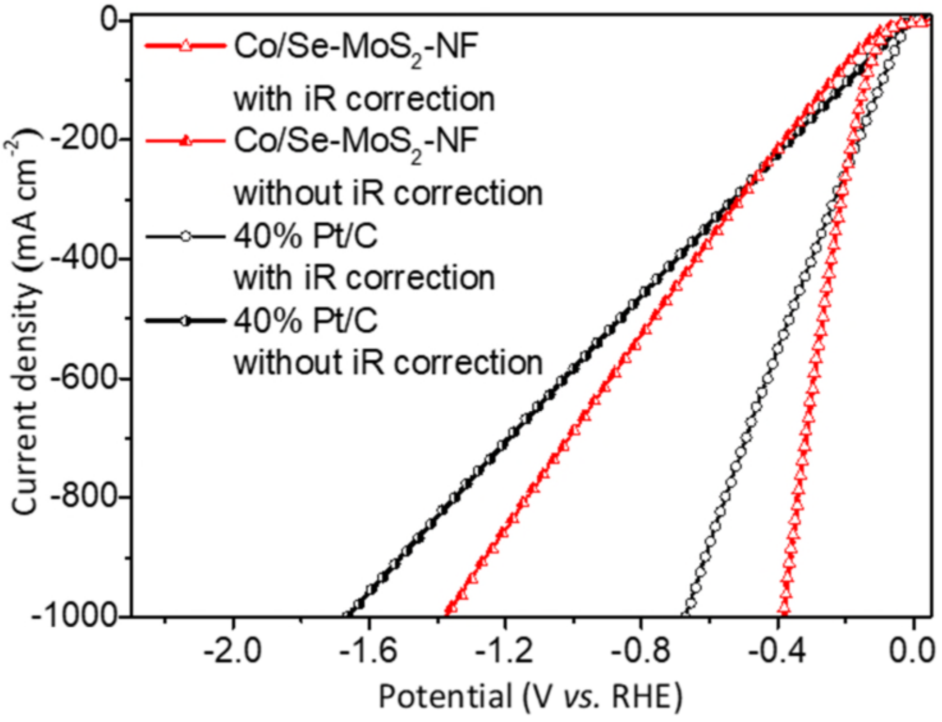

Supplementary Figure 21 | HER polarization curves with and without iR correction for the Co(10.4)/Se-MoS_2-NF and 40% Pt/C catalysts loaded on carbon paper.

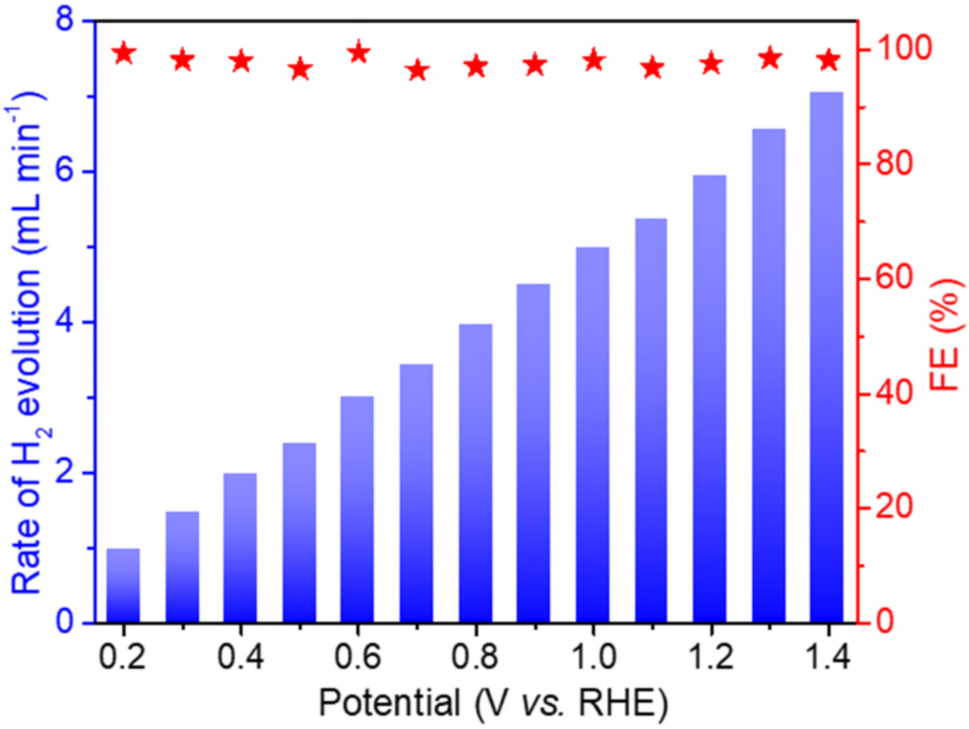

Supplementary Figure 22 | The production rate of H_2 and the corresponding faradaic efficiency at various working potentials using the Co(10.4)/Se-MoS_2-NF catalyst loaded on carbon paper.

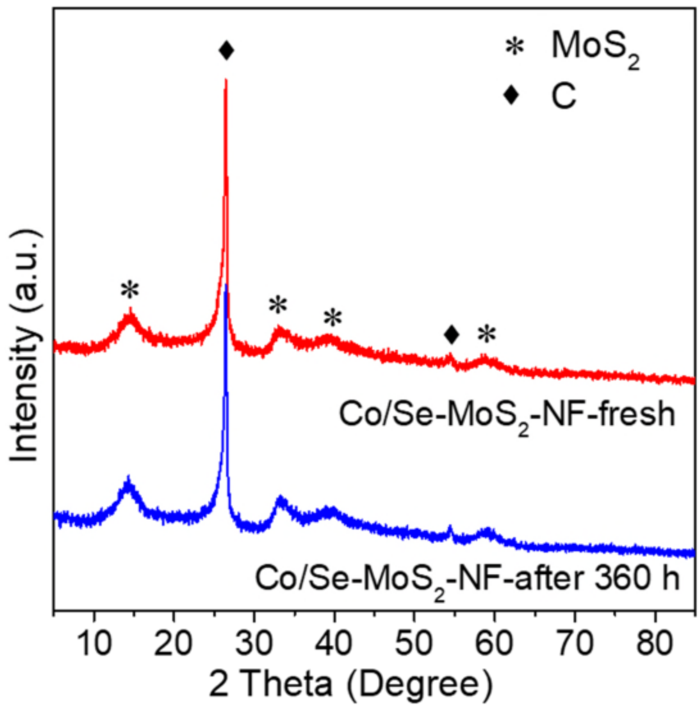

Supplementary Figure 23 | XRD patterns of the fresh Co(10.4)/Se-MoS_2-NF and that after 360 hours of durability test.

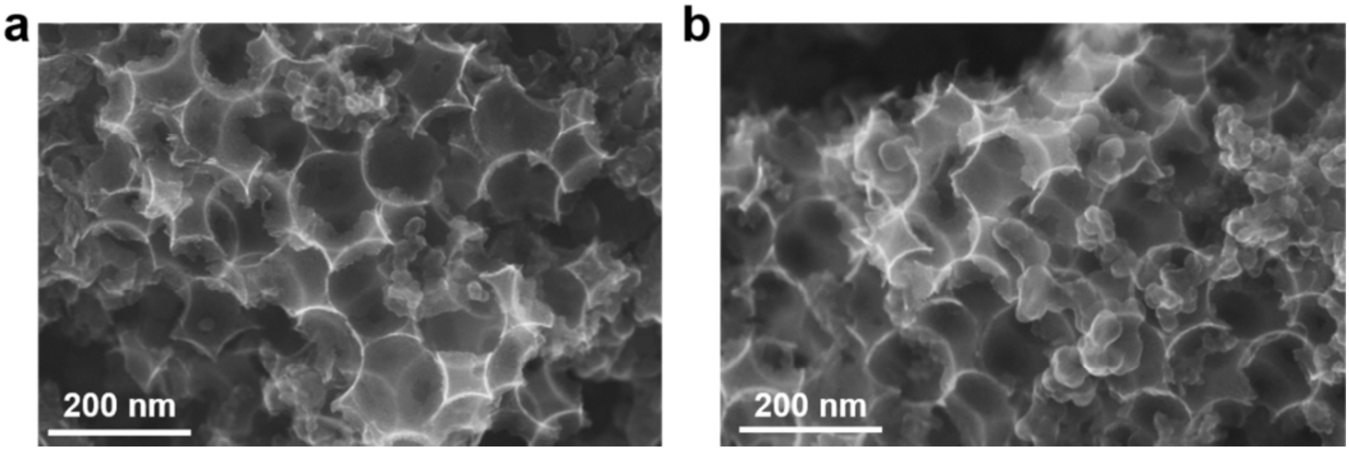

Supplementary Figure 24 | a,**b**, SEM images of the fresh Co(10.4)/Se-MoS_2-NF (**a**) and that after 360 hours of durability test (**b**).

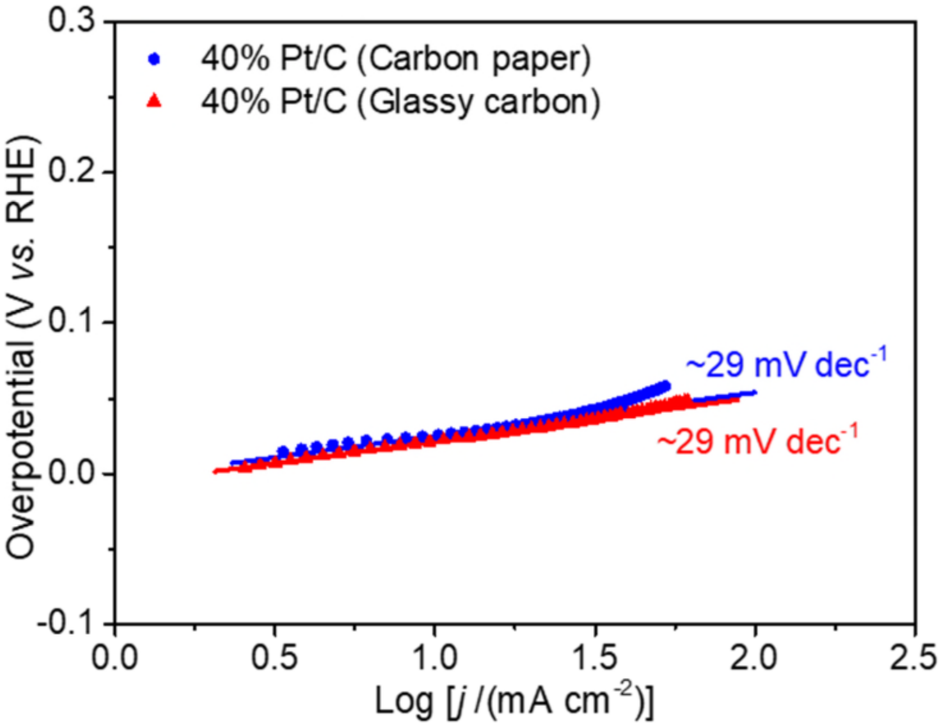

Supplementary Figure 25 | The Tafel slope plots of 40% Pt/C loading on the carbon paper in comparison with 40% Pt/C loading on the glassy carbon electrode.

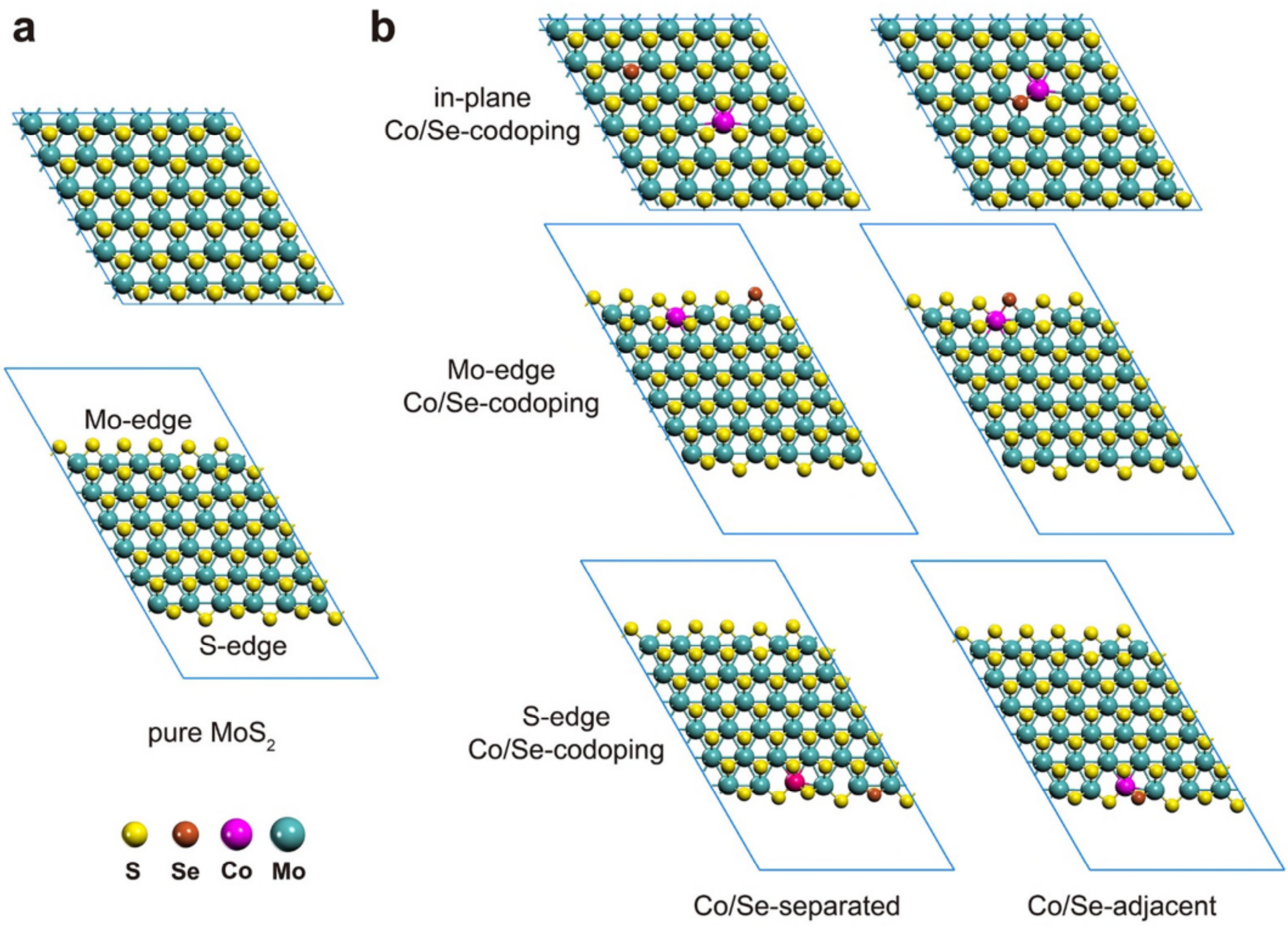

Supplementary Figure 26 | The supercell structures for modeling the pure MoS_2 basal plane and edges (**a**), and the Co/Se-codoped MoS_2 basal plane, Mo-edge, and S-edge with doping configurations of Co/Se-separated and Co/Se-adjacent (**b**).

Supplementary Table 1 | Lattice parameters of the Se-MoS_2-NF and MoS_2-NF samples derived from the XRD data using the software Jade6.

Sample	*a*/*b* (Å)	*c* (Å)
MoS_2-NF	3.152	12.47
Se(2.8)-MoS_2-NF	3.152	12.54
Se(5.7)-MoS_2-NF	3.151	12.53
Se(9.1)-MoS_2-NF	3.153	12.45
Se(10.3)-MoS_2-NF	3.152	12.51
Se(12.9)-MoS_2-NF	3.152	12.46

Supplementary Table 2 | Electrochemical impedance parameters obtained by simulating the Nyquist plots to the equivalent circuit model in Fig. 4c and Supplementary Fig. 8.

Sample	R_s [Ω]	CPE_1 [F]	R_1 [Ω]	CPE_{ct} [F]	R_{ct} [Ω]	n_1	n_2
Co(10.4)/Se-MoS_2-NF	5.51	0.005212	1.71	0.005378	7.196	0.8042	0.9336
Se(9.1)-MoS_2-NF	5.492	0.01224	0.3076	0.006521	11.58	0.8	0.8
Co(10.4)-MoS_2-NF	5.32	0.001759	0.3067	0.00269	23.85	0.8	0.8
MoS_2-NF	5.495	0.02434	0.09948	0.004167	44.08	0.8	0.8

Supplementary Table 3 | HER performances of heteroatom-doped MoS_2 catalysts in 0.5 M H_2SO_4.

Catalyst	Counter electrode	Overpotential at 10 mA cm^{-2} (mV)	Overpotential at 1000 mA cm^{-2} (mV)	Reference
Co(10.4)/Se-MoS_2-NF	graphite rod	104	382	This work
NP-MoS_2/CC	graphite rod	116	~480	1
MoS_2/ CNF-PHH-U	graphite rod	~150	450	2
MoS_2	graphite rod	~190	~570	3
Zn@MoS_2	platinum wire	194	N/A	4
N-MoS_2/CN	graphite rod	114	N/A	5
Cu@MoS_2	graphite rod	131	N/A	6
P, Se-MoS_2/CNTs	graphite rod	110	N/A	7
Pt-1T' MoS_2	graphite rod	180	N/A	8
$Re_{0.55}Mo_{0.45}S_2$	platinum/ graphite rod	147	N/A	9
Co-$^{S}MoS_2$	platinum coil	220	N/A	10
$Mo_{0.5}W_{0.5}S_2$	graphite rod	138	N/A	11
MCM@MoS_2-Ni	graphite rod	161	N/A	12
1% Pd-MoS_2	graphite rod	78	N/A	13
MoSSe	graphite rod	140	N/A	14
NF-MoS_2	platinum wire	220	N/A	15

Zn-MoS_2	graphite rod	~130	N/A	16
mPF-Co-MoS_2-16.7	graphite rod	156	N/A	17
Co-$MoSe_2$/NG	Pt plate	~180	N/A	18
CoMoS-2-C	Pt slice	135	N/A	19
Ni-Co-MoS_2	graphite rod	155	N/A	20
MoSSe@rGO	Pt mesh	153	N/A	21
P-1T-MoS_2	graphite rod	154	N/A	22
MoS_2Cl-VG	graphite rod	160	N/A	23
$V_{0.09}Mo_{0.91}S_2$	graphite rod	~230	N/A	24

Supplementary Table 4 | The experimental data of the vibrational frequencies, ZPE, integrated heat capacity ($\int_0^{298.15} C_p dT$), and entropy of the H_2 and H_2S molecules at 298.15 K from the NIST database, for calculating the free energy corrections. ΔG is calculated as ZPE + $\int_0^{298.15} C_p dT$ – TS, where T is 298.15 K.

Molecules	Vibrational Frequencies (cm^{-1})	ZPE (eV)	Integrated Heat Capacity from 0 to 298.15 K ($kJ\ mol^{-1}$)	S ($J\ mol^{-1}\ K^{-1}$)	ΔG (eV)
H_2	4161	0.26	8.47	130.68	-0.06
H_2S	2615, 2626, 1183	0.40	9.96	205.81	-0.13

Supplementary References

1 Sun, K. *et al.* Design of basal plane active MoS_2 through one-step nitrogen and phosphorus co-doping as an efficient pH-universal electrocatalyst for hydrogen evolution. *Nano Energy* **58**, 862-869 (2019).

2 Zhang, Z. *et al.* Controllable edge exposure of MoS_2 for efficient hydrogen evolution with high current density. *ACS Appl. Energy Mater.* **1**, 1268-1275 (2018).

3 Luo, Y. *et al.* Morphology and surface chemistry engineering toward pH-universal catalysts for hydrogen evolution at high current density. *Nat. Commun.* **10**, 269 (2019).

4 Wu, W. *et al.* Activation of MoS_2 basal planes for hydrogen evolution by zinc. *Angew. Chem. Int. Ed.* **58**, 2029-2033 (2019).

5 Wang, H. *et al.* Structural and electronic optimization of MoS_2 edges for hydrogen evolution. *J. Am. Chem. Soc.* **141**, 18578-18584 (2019).

6 Ji, L. *et al.* One-pot synthesis of porous 1T-phase MoS_2 integrated with single-atom Cu doping for enhancing electrocatalytic hydrogen evolution reaction. *Appl. Catal. B-Environ.* **251**, 87-93 (2019).

7 Zhu, T. *et al.* P,Se-codoped MoS_2 nanosheets as accelerated electrocatalysts for hydrogen evolution. *ChemCatChem* **11**, 689-692 (2019).

8 Wu, C. *et al.* Monoatomic platinum-anchored metallic MoS_2: correlation between surface dopant and hydrogen evolution. *J. Phys. Chem. Lett.* **10**, 6081-6087 (2019).

9 Yang, S.-Z. *et al.* Rhenium-doped and dtabilized MoS_2 atomic layers with basal-plane catalytic activity. *Adv. Mater.* **30**, 1803477 (2018).

10 Lau, T. H. M. *et al.* Transition metal atom doping of the basal plane of MoS_2 monolayer nanosheets for electrochemical hydrogen evolution. *Chem. Sci.* **9**, 4769-4776 (2018).

11 Wang, H. *et al.* Optimizing MoS_2 edges by alloying isovalent w for robust hydrogen evolution

activity. *ACS Catalysis* **8**, 9529-9536 (2018).

12 Zhang, H. *et al.* Surface modulation of hierarchical MoS_2 nanosheets by Ni single atoms for enhanced electrocatalytic hydrogen evolution. *Adv. Funct. Mater.* **28**, 1807086 (2018).

13 Luo, Z. *et al.* Chemically activating MoS_2 via spontaneous atomic palladium interfacial doping towards efficient hydrogen evolution. *Nat. Commun.* **9**, 2120 (2018).

14 Tan, C. *et al.* Preparation of high-percentage 1T-phase transition metal dichalcogenide nanodots for electrochemical hydrogen evolution. *Adv. Mater.* **30**, 1705509 (2018).

15 Wang, Y. *et al.* Fluorine- and nitrogen-codoped MoS_2 with a catalytically active basal plane. *ACS Appl. Mater. Inter.* **9**, 27715-27719 (2017).

16 Shi, Y. *et al.* Energy level engineering of MoS_2 by transition-metal doping for accelerating hydrogen evolution reaction. *J. Am. Chem. Soc.* **139**, 15479-15485 (2017).

17 Deng, J. *et al.* Multiscale structural and electronic control of molybdenum disulfide foam for highly efficient hydrogen production. *Nat. Commun.* **8**, 14430 (2017).

18 Ou, M. *et al.* Hydrothermal synthesis of few-layer and edge-rich cobalt-doped molybdenum selenide/nitrogenated graphene composite and investigation of its electrocatalytic activity for hydrogen evolution reaction. *Nano* **11**, 1650107 (2016).

19 Dai, X. *et al.* Co-doped MoS_2 nanosheets with the dominant CoMoS phase coated on carbon as an excellent electrocatalyst for hydrogen evolution. *ACS Appl. Mater. Inter.* **7**, 27242-27253 (2015).

20 Yu, X.-Y. *et al.* Formation of Ni-Co-MoS_2 nanoboxes with enhanced electrocatalytic activity for hydrogen evolution. *Adv. Mater.* **28**, 9006-9011 (2016).

21 Konkena, B. *et al.* MoSSe@reduced graphene oxide nanocomposite heterostructures as efficient and stable electrocatalysts for the hydrogen evolution reaction. *Nano Energy* **29**, 46-53 (2016).

22 Yin, Y. *et al.* Contributions of phase, sulfur vacancies, and edges to the hydrogen evolution reaction

catalytic activity of porous molybdenum disulfide nanosheets. *J. Am. Chem. Soc.* **138**, 7965-7972 (2016).

23 Zhang, X. *et al.* Amorphous MoS_xCl_y electrocatalyst supported by vertical graphene for efficient electrochemical and photoelectrochemical hydrogen generation. *Energy Environ. Sci.* **8**, 862-868 (2015).

24 Sun, X. *et al.* Semimetallic molybdenum disulfide ultrathin nanosheets as an efficient electrocatalyst for hydrogen evolution. *Nanoscale* **6**, 8359-8367 (2014).
