# Supplementary material for: Boosting hydrogen evolution on MoS2 via co-confining selenium in surface and cobalt in inner layer
Source: Nat Commun. 2020 Jul 3;11:3315. doi: 10.1038/s41467-020-17199-0 (PMC7334232; doi:10.1038/s41467-020-17199-0)
Supplement: Supplementary file 2 — Peer Review File [file 41467_2020_17199_MOESM2_ESM.docx]

Peer Review Information

**Journal:** Nature Communications

**Manuscript title:** Boost hydrogen evolution on MoS2 via co-confining selenium in surface and cobalt in inner layer

**Corresponding author name(s):** Prof Dehui Deng

**Editorial notes:**

**Transferred manuscripts**

This manuscript has been previously reviewed at another journal that is not operating a transparent peer review scheme. This document only contains reviewer comments and rebuttal letters for versions considered at *Nature Communications*.

**Reviewer comments & decisions:**

**Reviewer comments, first version:**

Reviewer #1 (Remarks to the Author):

This paper presents a very interesting experimental and modeling study of Co and Se co-doped MoS2 nanofoams. I will focus only on the modeling part, this being my expertise.

The DFT studies are interesting but not sufficiently complete in their description, presentation, and interpretation.

1) The authors have used a 6x6 nanoribbon of MoS2 to model the S-edge (with 75% S-coverage) and Mo-edge (with 50% S-coverage). There is clearly a built in dipole between these asymmetric edges, which is further perturbed by H adsorption. No mention has been made of dipole corrections in the DFT modeling (along the width of the ribbon) and this is likely to be important. If corrections were used, the authors need to clarify this in the methods; if not, they need to check whether the dipole corrections have any significant effect on the reported DFT energies or not.

2) In Figure 5(c), authors have shown that surface energy is negative for Se/Co-doped Mo- and S-edges and they suggest that this indicates stabilization of the structure relative to MoS2. First, it would be useful to have exact definitions of how these surface and edge energies are calculated. Second, as these structures are non-stoichiometric, the surface/edge energies are dependent on chemical potentials with at least one chemical potential being variable. (See for example, older papers by Northrup on III-V surfaces.) Third, what is the *absolute* stability of these surfaces/edges? If the surface or edge energy -- absolute, not relative, energy -- becomes negative, this indicates a surface/edge instability (leading to reconstruction). This also needs to be clarified.

3) Please clarify how hydrogen coverage at the edges is defined -- per Mo atom/S or Se binding site/...

4) Free-energies are reported to be calculated as E_total+ZPE+H-TS. There appears to be double counting here as enthalpy already contains internal energy in its definition (H=U+PV).

5) What were the exact PAW potentials used here? VASP provides various versions for an element -- with/without semi-core states, hard-vs-soft core, etc.


Reviewer #2 (Remarks to the Author):

The manuscript describes the synthesis and characterization of MoS2 foams and their derivatives: Co and Se doped structures. The structures are tested as catalysts for HER in acidic conditions in high current densities. DFT calculations are provided for the deltaGH.

I have the following comments:

1. Please switch discussing %wt to using %at, which allows better understanding of the actual fraction of S substituted for Se.

2. Such large substitional Se fractions should be evident as unit cell expansion according to Vegard’s law. Please provide the unit cell dimensions (a, c) next to the XRD patterns in the supporting. If there is a deviation from the expected values, please comment and explain.

3. Tuning the activity of MoS2 using Se by the electronegativity difference is presented in Nanoscale, 2018, 10, 16211–16216 and others. Here also, the non-linear effect of Se doping should be addressed – why do the authors think that a maximum activity is produced with 11.6% Se? What is the effect that leads to loss in activity beyond this doping ratio? Please discuss this feature with regards to the published data in the literature.

4. The TEM images do not provide clear evidence that the number/fraction/availability of edge sites is larger in MoS2+Se. Not only the authors only provide one image and not statistics, I do not see in the image a significant change in structure. I do not think that their statement is incorrect, but the microscopy data does not support it, while the XPS analysis, for example, is more convincing. I suggest the authors drop this conclusion from the TEM data, which otherwise is done skillfully and impressively.

5. Regarding the formation of CoSe, I did not see that it was considered in the EXAFS. Please also comment about the Co oxidation state in the structure. In addition, there is literature regarding Co doped MoSe2 and MoS2, and the authors should address their results and conclusions within the context of available data inside the main text, and also in addition update their table in the supporting.

6. The formation energy of the edges, mentioned in Figure 1f, please provide also absolute numbers and not just the difference.

7. Line 117-118- From the reduced Rct value for Se(11.6)-MoS2-NF the authors conclude an improved conductivity. This is a wrong conclusion as Rct from EIS measurements reflects the charge transfer resistance at the interface between catalysts and the electrolyte. Please correct. Plotting log(1/Rct) vs. η (where Rct is the charge transfer resistance) from the electrochemical impedance spectroscopy (EIS) data is another method to obtain the Tafel slope. It is suggested that the latter method can purely reveal the charge transfer kinetics, while Tafel slopes obtained from the polarization curves (LSV) may include contributions from the catalyst resistance (see Chem. Commun., 2013, 49, 8985; Nanoscale, 2017, 9, 13998). The authors should compare between these values (the Tafel slopes should be similar in the case of conductive materials) in order to validate their conclusion (or to measure conductivity in other direct methods.)

8. Please provide all the Tafel slopes for the data in Figure 2.

9. In Figure 4, the authors show HER activity for 2 different samples: drop casted on glassy carbon and on carbon paper. Specifically, the Tafel slope of the Pt/C of the carbon paper shows very high Tafel slope. First, please provide the Tafel slopes for the measurements in Figure 4D and comment to why this Pt is so low in activity compared to the Pt on panel b pf the same figure. It is clear that by comparing the optimal electrode to a degraded Pt electrode, one gets the wrong impression of the improvement.

10. Second, I do not understand why the authors show these two sets of measurements at all, and I think it is required to compare the values obtained at 10 and 100 mA/cm2 in these two samples, preferably providing only one set of data for the lower current densities as well as for the high current density.

11. The authors perform iR correction which is of course more prominent in the higher current densities. Please provide the overpotential numbers with and without iR correction for the current densities of 10, 100, 1000 mA/cm2. Second, please include in Figure S15 the full correction to 1000 mA/cm and state if this is the sample on carbon paper or glassy carbon in the caption.

12. Figure S19 shows the sample after durability test. Please provide evidence that there were no Pt depositions from the Pt mesh. The authors indeed show the use of an H cell, but this can also be verified directly.

13. Looking at the DFT calculations, it seems that the main conclusion is that Se within the basal plane stabilizes Co at the basal plane. Other effects are minor and seem to be changing with coverage and specific edge (and this is when corrugated edges such that appear in the TEM images were not even considered). If this is the case, I think the DFT section is quite too extended and with no focus and I recommend to revise it. In addition, the emphasize on the edges in the previous sections is not as important, since with Co doping the activation os of the basal plane. The authors need to work on the text such that a single and coherent message is provided.

14. I think the title is also ambiguous and I am not sure at all what is “confining inner-layer Co and surface Se atoms“. Please revise to indicate directly what you mean.


Reviewer #3 (Remarks to the Author):


The manuscript by Zheng et al. deals with the preparation of Co and Se doped MoS2 for the production of hydrogen (HER) via water electrolysis. The authors report the synthesis of MoS2 nanofoams using SiO2 nanoparticles as porous agent. The adjunction of Se and Co considerably improves the HER performance towards HER. The materials are very well characterized using various techniques including STEM and EXAFS. The electrocatalytic performances are correctly assessed using linear voltamperometry and potentiostatic measurements. Co/Se-MoS2-NF displays impressive activity with low overpotential and ultra-high current density up to 1 A/cm2 at overpotential < 0.4 V vs. RHE. Importantly, the performance is also found stable over 350 hours without any noticeable loss of activity.
Overall the manuscript is of high quality and could be published at Nature Communication. I have however an important remark. There are already several examples of similar materials reported in the literature (See for example: DOI: doi.org/10.1038/s41467-019-12997-7
). A careful comparison reveal that the present performances are lower than those from literature, probably because of the low electrical conductivity of the 2H phase of the nanosheets. The authors should emphasize the novelty of their proposed strategy in order to clearly demonstrate the novelty of their approach.

**Author rebuttal, first version:**

Reviewer # 1 (Remarks to the Author):

*This paper presents a very interesting experimental and modeling study of Co and Se co-doped MoS2 nanofoams. I will focus only on the modeling part, this being my expertise. The DFT studies are interesting but not sufficiently complete in their description, presentation, and interpretation.*

***Response:*** We appreciate your valuable comments and suggestions. We have revised the manuscript to further complete the DFT studies according to your comments.

***Comment 1.*** *The authors have used a 6x6 nanoribbon of MoS2 to model the S-edge (with 75% S-coverage) and Mo-edge (with 50% S-coverage). There is clearly a built in dipole between these asymmetric edges, which is further perturbed by H adsorption. No mention has been made of dipole corrections in the DFT modeling (along the width of the ribbon) and this is likely to be important. If corrections were used, the authors need to clarify this in the methods; if not, they need to check whether the dipole corrections have any significant effect on the reported DFT energies or not.*

***Response:*** We apologize for missing this important information in the methods part. We used dipole corrections in our DFT calculations. We have added this point in the methods part of the revised manuscript according to your suggestion (lines 480-481).

***Comment 2.*** *In Figure 5(c), authors have shown that surface energy is negative for Se/Co-doped Mo- and S-edges and they suggest that this indicates stabilization of the structure relative to MoS2. First, it would be useful to have exact definitions of how these surface and edge energies are calculated. Second, as these structures are non-stoichiometric, the surface/edge energies are dependent on chemical potentials with at least one chemical potential being variable. (See for example, older papers by Northrup on III-V surfaces.) Third, what is the *absolute* stability of these surfaces/edges? If the surface or edge energy -- absolute, not relative, energy -- becomes negative, this indicates a surface/edge instability (leading to reconstruction). This also needs to be clarified.*

***Response:*** Thank you very much for the comments. The surface energies were

calculated by using the free energies of H2, H2S, H2Se gas molecules, pristine MoS2, and Co bulk as the references. The chemical potential of each element is calculated as: μ(H) = 1/2 G(H2)

μ(S) = G(H2S) – G(H2)
μ(Se) = G(H2Se) – G(H2)
μ(Mo) = G(MoS2) – 2 μ(S)

μ(Co) = 1/2 G(Co-bulk) *

* the supercell of hcp Co bulk contains two Co atoms.

Then the absolute surface energy of a structure Mo*x*Co*y*S*z*Se*m* is given by:

γ = Etot(Mo*x*Co*y*S*z*Se*m*) – *x* μ(Mo) – *y* μ(Co) – *z* μ(S) – *m* μ(Se).

The absolute surface energies of the studied structures are all positive according to the above equations. They are all stable structures. According to the reviewer’s suggestions, we have added these descriptions in the methods part of the revised manuscript (lines 488-497).

***Comment 3.*** *Please clarify how hydrogen coverage at the edges is defined -- per Mo atom/S or Se binding site/...*

***Response:*** The hydrogen coverage at the Mo-edge is defined according to the terminating S or Se monomer sites, while that at the S-edge is defined according to the terminating S or Se dimer sites which are the active sites for the HER. We have added these definitions in the caption of Figure 5a in the revised manuscript accordingly (lines 264-266).

***Comment 4.*** *Free-energies are reported to be calculated as E_total+ZPE+H-TS. There appears to be double counting here as enthalpy already contains internal energy in its definition (H=U+PV).*

***Response:*** For gas phase molecules, H includes the parts of translational, vibrational, and PV contributions to the enthalpy. For the surface species, H includes only the part

of vibrational contribution to the enthalpy. Internal energy U is not included in the H here. To avoid confusions, we have revised related descriptions in the methods part to further clarify the definition of free energies (lines 483-484).

***Comment 5.*** *What were the exact PAW potentials used here? VASP provides various versions for an element -- with/without semi-core states, hard-vs-soft core, etc.*

***Response:*** The standard version of PAW pseudopotentials without using semi-core states were used for all elements. We have also tried POTCARs with s+p semis-core sates for Mo and Co and POTCAR with hard core for S in several calculations, in which the adsorption energies of H are almost unchanged (± 0.02 eV) and thus our results are not affected. To clarify this point, we have added the description in the methods part of revised manuscript (lines 472-473).

Reviewer #2 (Remarks to the Author):

*The manuscript describes the synthesis and characterization of MoS2 foams and their derivatives: Co and Se doped structures. The structures are tested as catalysts for HER in acidic conditions in high current densities. DFT calculations are provided for the deltaGH.*

*I have the following comments:*

***Comment 1.*** Please switch discussing %wt to using %at, which allows better understanding of the actual fraction of S substituted for Se.

***Response:*** Thank you for the comment. According to your suggestion, we have switched the weight percent into atomic percent for both the Co and Se atoms in all the doped structures and have revised manuscript accordingly.

***Comment 2.*** *Such large substitutional Se fractions should be evident as unit cell expansion according to Vegard’s law. Please provide the unit cell dimensions (a, c) next to the XRD patterns in the supporting. If there is a deviation from the expected values, please comment and explain.*

***Response:*** Thank you very much for the comment. We have calculated the unit cell dimensions (a, c) of the Se-MoS2-NF samples using the software Jade6 based on the XRD data, which show similar values compared with those of the pure MoS2-NF (Table R1). Since the atomic percentages of Se in the Se-MoS2-NF samples is still low, no obvious expansion in the a and c directions of the unit cell is observed in comparison to the pure MoS2-NF. We have added these data as Supplementary Table S1 and added related discussion in the revised manuscript accordingly (lines 71-72).

**Table R1.** Lattice parameters of the Se-MoS2-NF and MoS2-NF samples derived from

| the XRD data using the software Jade6. |  |  |
| --- | --- | --- |
| Sample | a, b (Å) | c (Å) |
| MoS2-NF | 3.152 | 12.47 |
| Se(2.8)-MoS2-NF | 3.152 | 12.54 |
| Se(5.7)-MoS2-NF | 3.151 | 12.53 |
| Se(9.1)-MoS2-NF | 3.153 | 12.45 |
| Se(10.3)-MoS2-NF | 3.152 | 12.51 |
| Se(12.9)-MoS2-NF | 3.152 | 12.46 |

***Comment 3.*** *Tuning the activity of MoS2 using Se by the electronegativity difference is presented in Nanoscale, 2018, 10, 16211–16216 and others. Here also, the non-linear effect of Se doping should be addressed – why do the authors think that a maximum activity is produced with 11.6% Se? What is the effect that leads to loss in activity beyond this doping ratio? Please discuss this feature with regards to the published data in the literature.*

***Response:*** Thank you very much for providing the reference which also reports that doping an appropriate content of Se into MoS2 can achieve a relatively optimum HER activity. In our work, a volcano-shaped relationship between the Se doping contents and HER activity is obtained for the Se-MoS2-NF samples, of which the one with 9.1% Se-doping content in atomic percentage (11.6% wt.) locates at the volcanic peak and shows the highest HER activity. To understand why over-doping of Se leads to reduced HER activity of MoS2, we have conducted additional DFT calculations of the effect of Se-doping on the edge reactivity. On the basis of Se-terminated edges, further substitution of S atoms by Se atoms at the Mo-edge side is energetically more favorable by 0.13 eV per Se compared with that at the S-edge side (Figure R2a), thus we select the Mo-edge to study the over-doping effect of Se. As more Se atoms are doped into the Se-terminated Mo-edge, the adsorption strength of H* at the edge Se sites is significantly weakened with much positive adsorption free energies (Figure R2b), which

consequently reduces the HER activity. Therefore, the loss of activity beyond the 9.1% doping ratio may be caused by the passivation of the Mo-edge due to excessive doping of Se. We have added these discussions in the revised manuscript according to your suggestion (lines 114-116, Supplementary Fig. S6).

**Figure R2 a.** Nanoribbon models of MoS2 for simulating the over-doping effect of Se on the edge reactivity. The red-dotted square labels the further substitution of S by Se

at the Se-terminated Mo- or S-edges. **b.** Adsorption free energies of H* (ΔGads(H*)) at the Se site of the Se-terminated Mo-edge with increasing doping content of Se.

***Comment 4.*** *The TEM images do not provide clear evidence that the number/fraction/availability of edge sites is larger in MoS2+Se. Not only the authors only provide one image and not statistics, I do not see in the image a significant change in structure. I do not think that their statement is incorrect, but the microscopy data does not support it, while the XPS analysis, for example, is more convincing. I suggest the authors drop this conclusion from the TEM data, which otherwise is done skillfully and impressively.*

***Response:*** Thank you for the valuable comments. We agree with you that the XPS and

EXAFS analysis are more convincing for evidencing the increase of edges in the Se-doped MoS2 than the TEM images. Thus, we have dropped this conclusion from the TEM data according to your suggestion and revised the manuscript accordingly (line 82).

***Comment 5.*** *Regarding the formation of CoSe, I did not see that it was considered in the EXAFS. Please also comment about the Co oxidation state in the structure. In addition, there is literature regarding Co doped MoSe2 and MoS2, and the authors should address their results and conclusions within the context of available data inside the main text, and also in addition update their table in the supporting.*

***Response:*** Thank you for the comments. The Fourier-transformed EXAFS spectra of the Co K-edge show that the Co atoms in the Co/Se-MoS2-NF are mainly coordinated by S atoms and the Co-S bond in Co/Se-MoS2-NF is shorter compared with that of the standard CoS sample (Figure 3g). A Co-Se bond (2.54 Å) is notably longer than a Co-S bond (2.41 Å) according to our DFT calculations but is not observed in the R-space of the Fourier-transformed EXAFS, which indicates that the CoSe phase should not be formed. The Co K-edge XANES spectra show that the adsorption edge of the MoS2-confined Co lies in between those of the CoS and Co3O4 (Supplementary Fig. S14). This indicates that the oxidation state of Co in the Co-MoS2-NF and Co/Se-MoS2-NF falls in between the valence of Co in the standard CoS and Co3O4 samples, implying that the Co atoms are coordinated by more S atoms than those in the CoS. Combined with the EXAFS results, this demonstrates that the Co atoms are covalently confined into the MoS2 lattice rather than being adsorbed on the surface or forming other hetero-phases such as CoS, CoSe and Co3O4.

We have checked some literatures regarding Co-doped MoS2 and MoSe2 (Nat. Commun. 2017, 8, 14430; ACS Appl. Mater. Interfaces 2015, 7, 27242-27253; Nano 2016, 11, 1650107), and then compared their HER activities with that of our Co(10.4)/Se-MoS2-NF catalyst, which shows that our Co(10.4)/Se-MoS2-NF catalyst possesses remarkably higher HER activity with a much lower overpotential. According to your suggestions, we have added these discussions in the revised manuscript and

updated the table in the revised Supplementary Information (lines 146-151, 192-193, Supplementary Table S3).

***Comment 6.*** *The formation energy of the edges, mentioned in Figure 1f, please provide also absolute numbers and not just the difference.*

***Response:*** According to your suggestion, we have provided the absolute formation energies of the S-terminated and Se/S-terminated edges in the revised Figure 1f and revised the manuscript accordingly (lines 105-106, Figure 1f).

***Comment 7.*** *Line 117-118- From the reduced Rct value for Se(11.6)-MoS2-NF the authors conclude an improved conductivity. This is a wrong conclusion as Rct from EIS measurements reflects the charge transfer resistance at the interface between catalysts and the electrolyte. Please correct. Plotting log(1/R_c_t) vs. η (where Rct is the charge transfer resistance) from the electrochemical impedance spectroscopy (EIS) data is another method to obtain the Tafel slope. It is suggested that the latter method can purely reveal the charge transfer kinetics, while Tafel slopes obtained from the polarization curves (LSV) may include contributions from the catalyst resistance (see Chem. Commun., 2013, 49, 8985; Nanoscale, 2017, 9, 13998). The authors should compare between these values (the Tafel slopes should be similar in the case of conductive materials) in order to validate their conclusion (or to measure conductivity in other direct methods.)*

***Response:*** Thank you for the constructive comments. We agree with you that the Rct from the EIS measurements reflects the charge transfer resistance at the interface between catalysts and the electrolyte. We have corrected our conclusion derived from the changes in the Rct values based on the EIS in the revised manuscript (lines 121-122).

According to your suggestion, we have also measured the EIS of different samples to obtain their Tafel slopes (Figure R3). Comparing the two groups of Tafel slopes derived from the EIS and LSV measurements, respectively, they show similar variation trend but have slight differences which stem from the electronic resistances of the catalysts (Chem. Commun., 2013, 49, 8985; Nanoscale, 2017, 9, 13998). The much

similar values of the EIS and LSV Tafel slopes for the Co(10.4)/Se-MoS2-NF sample indicates an improved conductivity of the catalyst. We have added these data as a new Supplementary Fig. S18 and revised the manuscript accordingly (lines 202-205).

**Figure R3.** Analysis of Tafel slopes from the EIS measurements of the MoS2-NF, Co(10.4)-MoS2-NF, Se(9.1)-MoS2-NF, Co(10.4)/Se-MoS2-NF and commercial 40% Pt/C.

***Comment 8.*** Please provide all the Tafel slopes for the data in Figure 2.

***Response:*** According to your suggestion, we have provided the Tafel slopes for the data in Figure 2a. As shown in Figure R4, the introduction of Se atoms leads to slight decrease in the Tafel slope of MoS2 ranging from 75 to 80 mV/dec. Thus, the HER over the Se-MoS2-NF samples follows the Volmer-Heyrovsky mechanism, being different from that over the Pt/C electrocatalyst via the Volmer-Tafel mechanism. We have added the data as a new Supplementary Fig. S7 and related discussion in the revised manuscript (lines 116-119).

**Figure R4.** The Tafel slope plots of the Se-MoS2-NF samples with different Se doping contents in comparison with those of the MoS2-NF and 40% Pt/C.

***Comment 9.*** *In Figure 4, the authors show HER activity for 2 different samples: drop casted on glassy carbon and on carbon paper. Specifically, the Tafel slope of the Pt/C of the carbon paper shows very high Tafel slope. First, please provide the Tafel slopes for the measurements in Figure 4D and comment to why this Pt is so low in activity compared to the Pt on panel b of the same figure. It is clear that by comparing the optimal electrode to a degraded Pt electrode, one gets the wrong impression of the improvement.*

***Response:*** Thank you for the comment. We have provided the Tafel slopes of the Pt/C catalyst loaded on carbon paper and glassy carbon electrode (Figure R5), which are almost the same (~29 mV/dec), thus indicating that their HER kinetics are identical. The overpotential at the current density of 10 mA cm^-2^ is 25 mV for the Pt/C catalyst drop-casted on carbon paper and is similar to that of 23 mV for the Pt/C drop-casted on glassy carbon, showing that their HER activities are close. Moreover, we conducted all the activity measurements of our catalysts and the Pt/C catalysts under identical conditions, thus their activities are comparable. To avoid confusion, we have added Tafel slopes of the samples loaded on carbon paper as Supplementary Fig. S25 and revised the manuscript accordingly (lines 439-440).

**Figure R5.** The Tafel slope plots of the 40% Pt/C and Co(10.4)/Se-MoS2-NF sample loaded on the carbon paper in comparison with that of the 40% Pt/C loaded on the glassy carbon electrode.

***Comment 10.*** *Second, I do not understand why the authors show these two sets of measurements at all, and I think it is required to compare the values obtained at 10 and 100 mA/cm^2^ in these two samples, preferably providing only one set of data for the lower current densities as well as for the high current density.*

***Response:*** Thanks for your comments. We employed the glassy carbon rotating disk electrode to perform HER measurements at low-level current densities of 10, 50, and 100 mA cm^-2^ since it is the standard and more accurate way. However, at ultra-high current densities of around 1000 mA cm^-2^ it is hard to obtain stable LSV curves using the glassy carbon rotating disk electrode due to the bubbling effect. Moreover, the glassy carbon rotating disk electrode cannot withstand the long-term stability test under high current densities. Thus, we loaded the catalyst on carbon paper to measure the HER activity and stability at large current densities up to 1000 mA cm^-2^. To clarify this point, we have added related descriptions in the methods part of the revised manuscript (lines 420-422, 438-439).

***Comment 11.*** *The authors perform iR correction which is of course more prominent in the higher current densities. Please provide the overpotential numbers with and without*

*iR correction for the current densities of 10, 100, 1000 mA/cm^2^. Second, please include in Figure S15 the full correction to 1000 mA/cm^-2^ and state if this is the sample on carbon paper or glassy carbon in the caption.*

***Response:*** Thank you for the helpful comments. The overpotentials at current densities of 10, 100, 1000 mA cm^-2^ with iR correction are 56, 141, and 382 mV for the Co/Se-MoS2-NF sample, and 25, 93, and 671 mV for the Pt/C catalyst, respectively; those without iR correction are 66, 241, and 1378 mV for the Co/Se-MoS2-NF sample, and 35, 193, and 1667 mV for the Pt/C catalyst, respectively (Figure R6). We have included the full correction to 1000 mA cm^-2^ in the Supplementary Fig. S21 and added the statement of using carbon paper in the caption according to your suggestions (lines 131, 230 and 234).

**Figure R6.** HER polarization curves with and without iR correction for the Co(10.4)/Se-MoS2-NF and 40% Pt/C catalysts loaded on carbon paper.

***Comment 12.*** *Figure S19 shows the sample after durability test. Please provide evidence that there were no Pt depositions from the Pt mesh. The authors indeed show the use of an H cell, but this can also be verified directly.*

***Response:*** Thanks for your comments. On the one hand, we employed a Nafion 117 membrane in the H-type electrochemical cell for separating the cathode and anode compartments so that the diffusion of Pt from the anode to cathode can be avoided

during measurements. On the other hand, according to your suggestion, we have performed inductively coupled plasma optical emission spectrometry (ICP-OES) measurement of the Co(10.4)/Se-MoS2-NF sample after the durability test, which shows that the content of Pt in the sample is below the detection limit, thus indicating that Pt is not likely deposited on the Co(10.4)/Se-MoS2-NF sample. We have added this point in the revised manuscript (lines 222-225).

***Comment 13.*** *Looking at the DFT calculations, it seems that the main conclusion is that Se within the basal plane stabilizes Co at the basal plane. Other effects are minor and seem to be changing with coverage and specific edge (and this is when corrugated edges such that appear in the TEM images were not even considered). If this is the case, I think the DFT section is quite too extended and with no focus and I recommend to revise it. In addition, the emphasize on the edges in the previous sections is not as important, since with Co doping the activation is of the basal plane. The authors need to work on the text such that a single and coherent message is provided.*

***Response:*** Thank you very much for your constructive comments. According our DFT calculations, Co-doping can not only activate the basal plane for the HER, but can also lead to over-activation of the edges with over strong adsorption of H* being harmful to the HER activity. Se-doping stabilizes both the Co-doped basal plane and edges, which on the one hand weakens moderately the adsorption of H* at both the in-plane and edge sites being more favorable for the HER activity, and on the other hand reduces the surface energy of the edges which favors the formation of abundant edges as active sites for the HER. These effects are independent to the coverage of H*. According to your suggestion, we have revised the manuscript and the Figure 5a to further clarify these points (lines 242-251).

***Comment 14.*** *I think the title is also ambiguous and I am not sure at all what is “confining inner-layer Co and surface Se atoms”. Please revise to indicate directly what you mean*.

***Response:*** Thank you for the comment. Since MoS2 is a sandwiched tri-layer atomic

structure, the expression was used to deliver the structural feature that the Co and Se are confined in the inner and outer layers substituting the Mo and S atoms, respectively. To further clarify this, we have revised the title to be “Boost hydrogen evolution on MoS2 via co-confining Se in surface and Co in inner” according to your suggestion.

Reviewer #3 (Remarks to the Author):

*The manuscript by Zheng et al. deals with the preparation of Co and Se doped MoS2 for the production of hydrogen (HER) via water electrolysis. The authors report the synthesis of MoS2 nanofoams using SiO2 nanoparticles as porous agent. The adjunction of Se and Co considerably improves the HER performance towards HER. The materials are very well characterized using various techniques including STEM and EXAFS. The electrocatalytic performances are correctly assessed using linear voltamperometry and potentiostatic measurements. Co/Se-MoS2-NF displays impressive activity with low overpotential and ultra-high current density up to 1 A/cm^2^ at overpotential < 0.4 V vs. RHE. Importantly, the performance is also found stable over 350 hours without any noticeable loss of activity.*

*Overall the manuscript is of high quality and could be published at Nature Communication. I have however an important remark. There are already several examples of similar materials reported in the literature (See for example: DOI:* [*doi.org/10.1038/s41467-019-12997-7)*](http://doi.org/10.1038/s41467-019-12997-7))*. A careful comparison reveal that the present performances are lower than those from literature, probably because of the low electrical conductivity of the 2H phase of the nanosheets. The authors should emphasize the novelty of their proposed strategy in order to clearly demonstrate the novelty of their approach.*

***Response:*** We appreciate your valuable comments on our work and we are also thankful to your recommendation of our manuscript for publication. The literature ([doi.org/10.1038/s41467-019-12997-7)](http://doi.org/10.1038/s41467-019-12997-7)) reported transformation of MoS2 from the 2H to 1T phase by loading atomic cobalt array onto the surface of MoS2 nanosheets, which exhibits a higher HER activity in acid electrolyte at low-level current densities below 100 mA cm^-2^. In our work, we proposed a novelty strategy of co-confining Co in the inner Mo layer and Se into the outer S layer of the MoS2, which significantly enhances both the HER activity and structural stability, achieving an ultra-high HER performance at industrial-level large-current-density of 1000 mA cm^-2^. We have emphasized this point in our revised manuscript and we have added the literature as a new Ref. 14 (Nat. Commun. 2019, 10, 5231).

**Reviewer comments, second version:**

Reviewer #1 (Remarks to the Author):

The authors have only partially addressed my comments on the DFT calculations. However, the response is still unclear and raises a few questions:

1) In response to my previous comment on edge energies/stability, the authors have provided a definition for \gamma, the surface energy ( Line 497). What is being called "surface energy" here is simply an energy excess -- also conventionally the "formation energy" -- and manifestly *not* related to the edge structure alone. The authors state in the response that "The absolute surface energies of the studied structures are all positive according to the above equations". Why is *absolute* energy of relevance? First, for a structure to be stable relative to its reference phases, the formation energy must be negative (thermodynamically preferable). Second, what is being reported here is not a surface energy -- it is simply an excess energy (as noted before) and involves any and all contributions including edges, substitutional defects, etc. Thus, I am not convinced that the edge models in Fig 5d are relevant -- these could still be unstable edges and thus irrelevant -- and I am certainly not convinced that the results in Fig 5c are meaningful.

2) The authors have added a note on the enthalpy calculation in response to my previous comment. Now that they have (partially) clarified their calculation, two points need to be made. First, to calculate the Gibbs free-energy of an adsorbed species, the conventional approach is to calculate the DFT (0 K) energy -- let's call this E -- and then add on the vibrational corrections as reflected in the zero-point energy (ZPE) and in the vibrational entropy (S_vib); S_vib can be calculated using a quantum harmonic oscillator model. In total then, G=E+ZPE-TS_vib. For gas phase molecules, it is well known (see papers by Karsten Reuter, Matthias Scheffler, for example) that an all-DFT approach does not give accurate results. Thus, it is conventional to use experimental gas-phase data, e.g., from the NIST database, and I would recommend that the authors do the same (unless they show that their DFT result is essentially the same as the experimental result). The authors should also provide detailed equations explaining how the free-energy is calculated -- two lines in the methods section are most inadequate and does not help others in reproducing their results.


Reviewer #2 (Remarks to the Author):

My comments and concerns were fully answered by the authors.

**Author rebuttal, second version:**

Reviewer #1 (Remarks to the Author):

*The authors have only partially addressed my comments on the DFT calculations. However, the response is still unclear and raises a few questions:*

***Response:*** We are grateful for your comments and suggestions, which have helped us to improve the quality of this manuscript. We have made detailed and point-to-point response to your comments and revised the manuscript accordingly with all changes being highlighted in yellow in the revised manuscript and Supplementary Information.

***Comment 1.*** *In response to my previous comment on edge energies/stability, the authors have provided a definition for \gamma, the surface energy ( Line 497). What is being called "surface energy" here is simply an energy excess -- also conventionally the "formation energy" -- and manifestly *not* related to the edge structure alone. The authors state in the response that "The absolute surface energies of the studied structures are all positive according to the above equations". Why is *absolute* energy of relevance? First, for a structure to be stable relative to its reference phases, the formation energy must be negative (thermodynamically preferable). Second, what is being reported here is not a surface energy -- it is simply an excess energy (as noted before) and involves any and all contributions including edges, substitutional defects, etc. Thus, I am not convinced that the edge models in Fig 5d are relevant -- these could still be unstable edges and thus irrelevant -- and I am certainly not convinced that the results in Fig 5c are meaningful.*

***Response*:** We thank you for the helpful comments. We agree with you that the surface energy in our work used the same definition with that of the formation energy. We used the term ‘surface energy’ in order to stress the stabilities of the doped/undoped MoS2 surfaces and edges. To avoid confusion, we have modified the ‘surface energy’ to be ‘formation energy’ in the revised manuscript.

The statement “*The absolute surface energies of the studied structures are all positive according to the above equations*” in the previous response was made owing to a misunderstanding of your previous comment 2 “*If the surface or edge energy -­absolute, not relative, energy -- becomes negative, this indicates a surface/edge instability (leading to reconstruction)*”. We apologize for this misinterpretation. Since stable references such as H2, H2S, H2Se, Co bulk, and intact MoS2 were used, the formation energies of the studied structures are all positive and we agree with you that this indicates only the stability of the structure relative to the reference phases and is not of relevance. In Figure 5c, we focus on illustrating the effect of Se-doping in stabilizing the Co-doped surface and edges. To make this point clear, we have conducted new calculations of Co/Se-codoped structures with different doping configurations of Co and Se, i.e. Co and Se being separated or adjacent to each other, and have remade the Figure 5c and 5d in which their relative formation energies and atomic structures are compared. A lower relative formation energy indicates a more stable doping configuration. The revised Figure 5c shows that the Co/Se-adjacent doping configuration is always more stable than the Co/Se-separated doping configuration either in the basal plane or at the Mo- and S-edges, which demonstrates that the Se-doping can stabilize the Co-doped surface and edges by forming Co-Se bonds. We have updated the Figure 5c and 5d (lines 260-261, 268-271) and revised related discussion (lines 253-256), and we have added the calculation details (lines 502­504) and the supercell models (Supplementary Figure S26) in the revised manuscript.

***Comment 2.*** *The authors have added a note on the enthalpy calculation in response to my previous comment. Now that they have (partially) clarified their calculation, two points need to be made. First, to calculate the Gibbs free-energy of an adsorbed species, the conventional approach is to calculate the DFT (0 K) energy -- let's call this E -- and then add on the vibrational corrections as reflected in the zero-point energy (ZPE) and in the vibrational entropy (S_vib); S_vib can be calculated using a quantum harmonic oscillator model. In total then, G=E+ZPE-TS_vib. For gas phase molecules, it is well known (see papers by Karsten Reuter, Matthias Scheffler, for example) that an all-DFT*

*approach does not give accurate results. Thus, it is conventional to use experimental gas-phase data, e.g., from the NIST database, and I would recommend that the authors do the same (unless they show that their DFT result is essentially the same as the experimental result). The authors should also provide detailed equations explaining how the free-energy is calculated -- two lines in the methods section are most inadequate and does not help others in reproducing their results.*

***Response*:** Thank you for your valuable comments and suggestions. We agree with you on the calculations of the free energies of the adsorbed species and gas-phase molecules. In our calculations of the free energies of the adsorbed species, we considered one more

*298.15*

term in addition to the E + ZPE - _TSvib_ parts, i.e. the Uvib = *rJ	cv(vib)dT* under the

*o*

harmonic oscillation approximation. For calculating the free energy corrections of gas-phase molecules, we agree with you that all-DFT approaches can hardly give accurate results. However, we have compared our DFT-calculated thermochemistry data (Table R1) with the experimental data from the NIST (Table R2) for the H2 and H2S molecules (the experimental data of the H2Se molecule is not complete and thus is not compared here), and we find that they are very close and the difference in the final free energy correction is negligible. Nevertheless, to avoid doubt on our data, we have chosen to use the experimental thermochemistry data for gas-phase molecules from the NIST database according to your suggestion. Since the experimental part of integrated heat capacity from 0 to 298.15 K for the H2Se molecule is not provided in the NIST database, we use solid phase MoSe2 instead of the H2Se molecule as the reference state of Se and have revised the manuscript accordingly (lines 492-493, 497). It should be noted that this does not change our results and conclusion. To further clarify our methods of calculating the free energies according to your suggestion, we have added more details in the DFT methods part (lines 485-491) and have added the Table R2 as a new Supplementary Table S4 in the revised manuscript.

**Table R1.** The DFT-calculated vibrational frequencies, ZPE, integrated heat capacity

.

(∫	), and entropy of gas-phase molecules at 298.15 K obtained by using the


Thermochemistry module of the Atomic Simulation Environment based on ideal gas

.

consumption. ΔG is calculated as ZPE + ∫	– TS, where T is 298.15 K.


| Molecules | DFT Calculated  Vibrational  Frequencies  (cm^-1^) | ZPE  (eV) | Integrated Heat  Capacity from 0 to  298.15 K  (eV) | S  (eV K^-1^) | ΔG  (eV) |
| --- | --- | --- | --- | --- | --- |
| H2  H2S | 4302.44  2658.39,  2646.82,  1177.76 | 0.27  0.40 | 0.091  0.104 | 1.41E-3  2.13E-3 | -0.06  -0.13 |

**Table R2**. The experimental data of the vibrational frequencies, ZPE, integrated heat

.

capacity (∫	), and entropy of the H2 and H2S molecules at 298.15 K from the


NIST database, for calculating the free energy corrections. ΔG is calculated as ZPE +

.

∫	– TS, where T is 298.15 K.


| Vibrational  Molecules Frequencies  (cm^-1^) | ZPE  (eV) | Integrated Heat  Capacity from 0 to  298.15 K  (kJ mol^-1^) | S  (J mol^-1^ K^-1^) | ΔG (eV) |
| --- | --- | --- | --- | --- |

H2	4161	0.26	8.47	130.68	-0.06

2615, 2626,

H2S	0.40	9.96	205.81	-0.13
1183

Reviewer #2 (Remarks to the Author):

*My comments and concerns were fully answered by the authors.*

***Response:*** Thank the reviewer for all the comments and suggestions which have helped

a lot in improving the quality of this manuscript.

**Reviewer comments, third version:**

Reviewer #1 (Remarks to the Author):

The authors have satisfactorily addressed all of my concerns. The paper may be accepted for publication.

**Author rebuttal, third version:**
